# The Depletion Mechanism Actuates Bacterial Aggregation by Exopolysaccharides and Determines Species Distribution & Composition in Bacterial Aggregates

**DOI:** 10.3389/fcimb.2022.869736

**Published:** 2022-06-16

**Authors:** Patrick R. Secor, Lia A. Michaels, DeAnna C. Bublitz, Laura K. Jennings, Pradeep K. Singh

**Affiliations:** ^1^ Division of Biological Sciences, University of Montana, Missoula, MT, United States; ^2^ Department of Microbiology, University of Washington, Seattle, WA, United States

**Keywords:** *Pseudomonas aeruginosa*, *Staphylococcus Aureus*, *Burkholderia*, aggregate, biofilm, quorum sensing, type VI secretion, antimicrobial tolerance

## Abstract

Bacteria in natural environments and infections are often found in cell aggregates suspended in polymer-rich solutions, and aggregation can promote bacterial survival and stress resistance. One aggregation mechanism, called depletion aggregation, is driven by physical forces between bacteria and high concentrations of polymers in the environment rather than bacterial activity *per se*. As such, bacteria aggregated by the depletion mechanism will disperse when polymer concentrations fall unless other adhesion mechanisms supervene. Here we investigated whether the depletion mechanism can actuate the aggregating effects of *Pseudomonas aeruginosa* exopolysaccharides for suspended (i.e. not surface attached) bacteria, and how depletion affects bacterial inter-species interactions. We found that cells overexpressing the exopolysaccharides Pel and Psl remained aggregated after short periods of depletion aggregation whereas wild-type and mucoid *P. aeruginosa* did not. In co-culture, depletion aggregation had contrasting effects on *P. aeruginosa’s* interactions with coccus- and rod-shaped bacteria. Depletion caused *S. aureus* (cocci) and *P. aeruginosa* (rods) to segregate from each other and *S. aureus* to resist secreted *P. aeruginosa* antimicrobial factors resulting in species co-existence. In contrast, depletion aggregation caused *P. aeruginosa* and *Burkholderia* sp. (both rods) to intermix, enhancing type VI secretion inhibition of *Burkholderia* by *P. aeruginosa*, leading to *P. aeruginosa* dominance. These results show that in addition to being a primary cause of aggregation in polymer-rich suspensions, physical forces inherent to the depletion mechanism can promote aggregation by some self-produced exopolysaccharides and determine species distribution and composition of bacterial communities.

## Introduction

Bacteria are often found within cell aggregates suspended in polymer-rich environments. Examples include bacteria growing in soil (Wilpiszeski et al., 2019), aqueous environments (Blom et al., 2010), and those living in animal host secretions such as mucus, pus, and sputum (Bjarnsholt et al., 2013; Kragh et al., 2014; Bay et al., 2018; Speare et al., 2020). Aggregated growth is thought important because it can increase the ability of bacteria to survive environmental stresses such as pH and osmotic extremes, as well as host-derived and pharmaceutical antimicrobials (Stewart and Costerton, 2001; Hall-Stoodley et al., 2004). Bacterial aggregation also affects phenotypes relevant to host-microbe interactions such as bacterial invasiveness, virulence factor production, resistance to predation by protozoans, and resistance to phagocytic uptake (Kharazmi, 1991; Hahn et al., 2000; Jesaitis et al., 2003; Alhede et al., 2011; Sonderholm et al., 2017).

Bacteria can aggregate *via* bridging aggregation, which occurs when adhesions, polymers, or other molecules bind cells to one another. For example, biofilm formation occurs when cells accumulate next to each other on surfaces and produce exopolysaccharides and other matrix components that enable them to stick together *via* bridging interactions (Costerton et al., 1995; Davey and O'Toole, 2000). However, bacteria suspended in solutions are less likely to accumulate immediately adjacent to each other by clonal growth because random (i.e. Brownian) movement or fluid flows will disperse them. This reduces the opportunity for cell-cell bridging interactions *via* self-produced exopolysaccharides.

**Figure 1:**
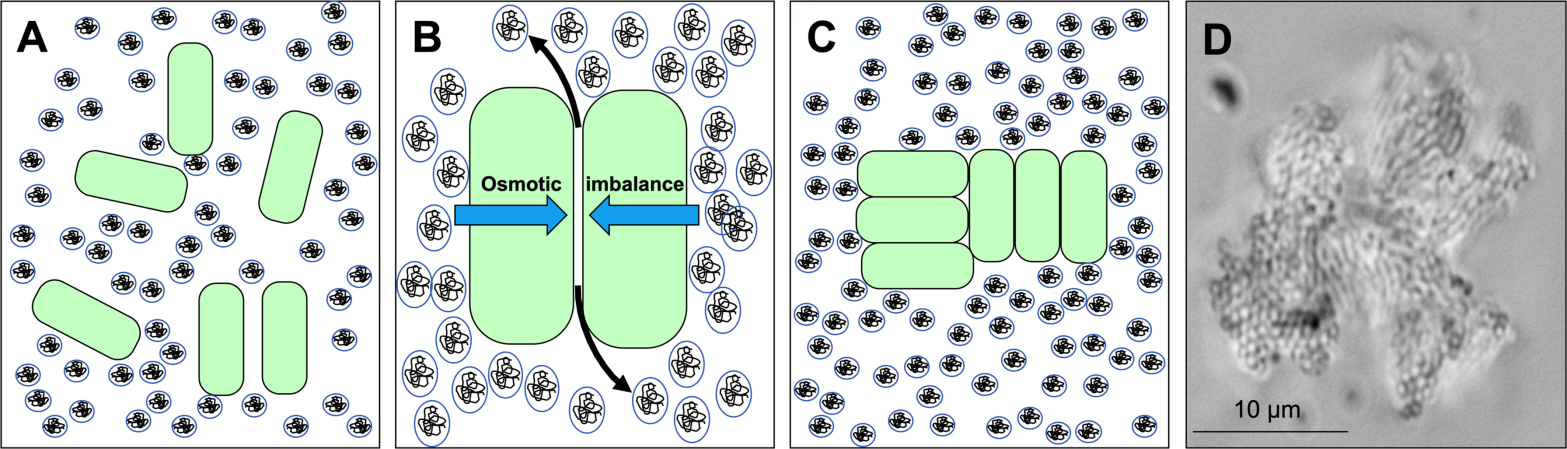
Depletion aggregation aggregates bacterial cells in environments crowded with non-adsorbing polymers. **(A)** Bacterial cells (green) are suspended in an environment with high concentrations of non-adsorbing polymer (circles). **(B)** Polymers in between cells are restricted in their conformational freedom and spontaneously move out from in between cells (black arrows), increasing their entropy. The polymer concentration gradient across the cells produces an osmotic imbalance (blue arrows). **(C)** The osmotic imbalance (i.e., the depletion force) physically holds the cells together in aggregates. **(D)** Representative image of a *P. aeruginosa* PAO1 depletion aggregate with PEG 35 kDa as the polymer.

Another general yet underappreciated mechanism is depletion aggregation (Marenduzzo et al., 2006). Depletion aggregation occurs in environments containing high concentrations of non-adsorbing polymers (Asakura and Oosawa, 1958; Poon, 2002). Such conditions exist in the cytoplasm of eukaryotic cells (Marenduzzo et al., 2006), mucosal surfaces (Preska Steinberg et al., 2019), cystic fibrosis (CF) airways (Secor et al., 2018), wounds (Clark, 1996), biofilm matrices (Dorken et al., 2012), and other settings. Depletion aggregation is initiated when bacteria spontaneously come into close contact with each other (**Figure 1A**). This causes the polymers in between cells to become restricted in their configurational freedom, which decreases their entropy. Polymers will spontaneously move out from in between cells (Schwarz-Linek et al., 2010) which results in a polymer concentration gradient across adjacent bacterial cells and an osmotic imbalance (i.e., the depletion force) that physically holds the cells together (**Figures 1B, C**) (Schwarz-Linek et al., 2010; Peters et al., 2021). A representative image of a *Pseudomonas aeruginosa* PAO1 depletion aggregate produced by exposure to the model polymer polyethylene glycol (PEG) is shown in **Figure 1D**.

Depletion aggregation is a spontaneous process driven by physical forces generated in environments with high concentrations of polymers. Thus, if bacteria and polymer concentrations are high enough, aggregation *via* depletion will occur as a default and obligatory outcome unless mechanisms like mechanical disruption or bacterial motility (Schwarz-Linek et al., 2012) produce stronger counteracting forces to disperse cells. Likewise, diluting the polymers will reduce the osmotic force holding the aggregates together and result in aggregate dispersal unless other cell-to-cell adhesion interactions supervene.

Previous work has shown that the concentrations of host-derived polymers like mucin, DNA, and F-actin found at infection sites can cause bacterial depletion aggregation in a similar manner to model polymers like PEG, and that depletion aggregation induces an antibiotic-tolerance phenotype in *P. aeruginosa* (Secor et al., 2018). Here we investigated whether the depletion mechanism can actuate durable cell-to-cell adhesion of suspended bacteria by the exopolysaccharides implicated in the formation of surface-attached *P. aeruginosa* biofilms. We also investigated how depletion aggregation affects interactions between bacterial species that are found together in some settings such as the infected CF lung.

## Materials and Methods

### Chemicals/Growth Media/Strains

Growth media (Lysogeny broth, LB), polyethylene glycol MW 2,000 and 35,000 Da, and antibiotics were purchased from Sigma. Strains and their sources are listed in **Table 1**.

**Table 1:** Strains used in this study.

Strain	Description	Source
*P. aeruginosa* PAO1	Wild type	(Holloway et al., 1979)
PAO1 Δ*pelA/pslBCD/algD*	Deletion of *pelA*, *pslB, pslC, pslD*, and *algD*	(Colvin et al., 2012)
PAO1 Δ*wspF*/*pslD*; pBAD::Pel	Deletion of *wspF* and *pslD*; arabinose-inducible Pel operon	(Colvin et al., 2013)
PAO1 Δ*wspF*/*pelF*; pBAD::Psl	Deletion of *wspF* and *pelF*; arabinose-inducible Psl operon	(Jennings et al., 2015)
MucA22 (PDO300)	A *mucA22* allele derivative of PAO1 constructed by allelic exchange	(Mathee et al., 1999)
PAO1 Δ*mucA*	Contains a truncated *mucA* allele	(Pritchett et al., 2015)
Clinical Isolates	*P. aeruginosa* clinical isolates from various patients	(Smith et al., 2006)
PAO1 Δ*lasR/rhlR*	Deletion of *lasR* and *rhlR*	(Siehnel et al., 2010)
PAO1 Δ*clpV1*	Deletion of *clpV1*	(Mougous et al., 2006)
PAO1 attTn7::*GFP*	Constitutive expression of GFP	(Choi and Schweizer, 2006)
PAO1 ΔclpV1; attTn7::*GFP*	Deletion of *clpV1*; constitutively expressing GFP	(LeRoux et al., 2012)
PAO1 attTn7:*TFP*	Constitutive expression of TFP	(Zhao et al., 2013)
PAO1 attTn7::*YFP*	Constitutive expression of YFP	(Zhao et al., 2013)
*E. coli* pUCP18-*mCherry*	Carries plasmid expressing IPTG-inducible mCherry	(Irie et al., 2012)
*B. thailandensis* E264	Wild type	(Yu et al., 2006)
*B. thailandensis* E264 attTn7::*mCherry*	Constitutive expression of mCherry	(LeRoux et al., 2012)
*B. cenocepacia* K56-2 attTn7::*GFP*	Constitutive expression of GFP	(Varga et al., 2013)
*S. aureus* SH1000	Wild type	(Horsburgh et al., 2002)
*S. aureus* pCE-SarA-*mCherry*	Constitutive expression of mCherry	(Malone et al., 2009)

### PEG-Induced Depletion Aggregation of Bacteria

For PEG-induced depletion aggregation, bacteria were added at the indicated densities to either LB diluted 4:6 with distilled water or LB diluted with 50% PEG 35 kDa (w/vol) prepared in distilled water to ensure that nutrient concentrations were the same in dispersed and aggregated conditions. LB was diluted with water or 50% w/vol PEG 35 kDa for all experiments described unless noted otherwise. Cultures were then incubated on a roller (60 rpm) at 37°C unless indicated otherwise.

### Aggregate Reversibility Assays

**Figure 2:**
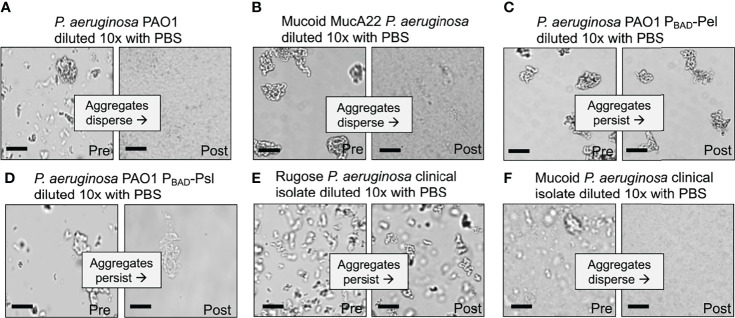
Depletion aggregate dispersal phenotypes of *P. aeruginosa* laboratory strains and CF clinical isolates. **(A–F)** Aggregate dispersal of the indicated strains and isolates was measured. Depletion aggregation was induced with 30% w/vol PEG 35 kDa for 18 hours. Depletion aggregates were then diluted 10X with PBS and representative images were acquired immediately pre- and immediately post-dilution. Scale bar 40 µm.

**Figure 3:**
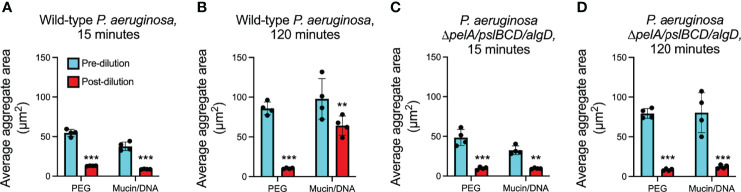
Depletion aggregate dispersal phenotypes of *P. aeruginosa* in PEG or mixtures of mucin/DNA. **(A, B)** Fluorescent *P. aeruginosa* PAO1 (wild type) or **(C, D)** the exopolysaccharide triple mutant Δ*pelA/pslBCD/algD* were aggregated by either PEG 35 kDa or a mixture of mucin and DNA. Fifteen- or 120-minutes post-aggregation, cultures were diluted 10X with PBS; fluorescent images were acquired immediately pre- and immediately post-dilution. The area of fluorescent aggregates was measured using Velocity image analysis software. The mean ± SD of at least 100 aggregates per replicate (n = 4) is shown, **P<0.01, ***P<0.001, Student’s *t*-test. See representative aggregate images in **Figure S3**.

The indicated bacterial strains in **Figure 2** were grown overnight in LB at 37°C with shaking. One hundred µl of overnight cultures were used to inoculate 3 ml of LB+PEG 35 kDa. After 18-h of growth, 100 µl of the indicated cultures were removed to a 1.5 ml tube containing 900 µl of either 1x PBS or PBS supplemented with 30% w/vol PEG 35 kDa and vortexed. PBS was used to facilitate imaging. Imaging was performed on 50 µl culture aliquots pre- and post-dilution using a Leica DM1000 LED microscope by spotting onto a glass slide. Aggregate dispersal was either scored i) by eye as either aggregated or dispersed by comparing to undiluted control cultures. Aggregate reversal assays shown in **Figure 3** were performed as follows: *P. aeruginosa* PAO1 or Δ*pelA/pslBCD/algD* constitutively expressing YFP were grown overnight in LB at 37°C, spun down, and washed and resuspended in PBS at 1x10^9^ CFU/ml. Bacteria were added at a 1:1 ratio to either an 8% (w/vol) solution of mucin (porcine gastric mucin) and 4 mg/ml DNA (HMW, salmon sperm DNA) in PBS or a 30% (w/vol) solution of PEG 35k in PBS. Cultures were incubated on a roller (60 rpm, 37°C) for 15 or 120 minutes. Bacteria (50 ul) were diluted with 200 ul PBS and mixed by inverting the tube. 20ul of diluted (or undiluted) cultures were placed onto a slide and bacteria were imaged (YFP) such that aggregates were bright and had a distinct defining border from any background. Aggregate area was calculated using Velocity’s (Improvision) find object tool using intensity and a minimum aggregate size of 16.64 µm^2^ (40 pixels). The mean are ± SD of at least 100 aggregates per replicate was then calculated.

### Bacterial Competition Assays

*S. aureus* SH1000 (Horsburgh et al., 2002) and *P. aeruginosa* PAO1 (Holloway et al., 1979) were grown overnight at 37°C with shaking in LB broth. *S. aureus* and *P. aeruginosa* were pelleted and resuspended at 10^8^ CFU/ml in fresh LB broth. One hundred µl of each culture was added to 2 ml LB supplemented with either 30% w/vol PEG (35 kDa or 2 kDa) where indicated. Bacteria were grown in co-culture for 18 h and viable bacteria were enumerated by serial dilution and plating on LB plates. For experiments investigating the effects of quorum-regulated antimicrobials on *S. aureus* killing, *P. aeruginosa* PAO1 or *ΔlasR/rhlR* (Siehnel et al., 2010) were grown overnight at 37°C with shaking in 50 ml LB broth in a 250 ml flask. Bacteria were removed by centrifugation (10 minutes, 9,000 x g) and supernatants were filter sterilized using bottle top vacuum filters with 0.2 µm pore size (Millipore). PEG 2 kDa or 35 kDa was added to these supernatants to a final concentration of 30% w/vol where indicated. *S. aureus* was inoculated into *P. aeruginosa* supernatants at 10^8^ CFU/ml and cultured for 6 h at 37°C on a roller at 60 rpm. Viable *S. aureus* were enumerated by serial dilution and plating onto LB agar plates. To investigate TSS mediated killing, *P. aeruginosa* PAO1, *ΔclpV1* (Mougous et al., 2006), and *B. thailandensis* E264 (Yu et al., 2006) were grown overnight at 37°C with shaking in LB broth. Bacteria were resuspended in fresh LB at 10^9^ CFU/ml. One hundred µl containing 1x10^8^ CFU *P. aeruginosa* PAO1 or Δ*clpV1* and 100 µl containing 2.0x10^7^ CFU *B. thailandensis* were added to 800 µl LB or the indicated polymer solutions and incubated in co-culture for 24 h at 37°C on a roller at 60 rpm. Viable bacteria were enumerated by serial dilution and plating on LB plates. For fluorescent imaging of aggregates, strains PAO1 or *ΔclpV1* constitutively expressing GFP (PAO1 attTn7::*GFP* (Choi and Schweizer, 2006),) were co-cultured with *B. thailandensis* E264 attTn7::*mCherry* for 24 hours (LeRoux et al., 2012).

### Fluorescent Microscopy

*S. aureus* SH1000 carrying the fluorescent reporter pCE-SarA-*mCherry* (Malone et al., 2009), *P. aeruginosa* PAO1 attTn7::*GFP*, PAO1 attTn7::*TFP* (Zhao et al., 2013), PAO1 attTn7::*YFP* (Zhao et al., 2013), *E. coli* carrying pUCP18-mCherry (Irie et al., 2012), *B. cenocepacia* K56-2 attTn7::*GFP* (Varga et al., 2013) and *B. thailandensis* E264 attTn7::*mCherry* were co-cultured as indicated. Depletion aggregates assembled from dead bacteria were prepared by washing and resuspending overnight cultures of PAO1 YFP or PAO1 TFP in PBS at a concentration of 10^9^ CFU/ml. Formaldehyde (16%, Thermo) was added slowly to bacteria while vortexing to a final concentration of 4% vol/vol. Bacteria were allowed to fix for 30 minutes with constant mixing to prevent bacteria from clumping. Cells were then centrifuged for 10 minutes at 9,000 x g, washed twice with PBS, and resuspended in 1 ml PBS. Complete bacterial killing was confirmed by plating fixed bacteria on LB agar. One hundred µl of the indicated fixed strains were added to 2 ml PBS or PBS+30% PEG 35 kDa. Bacteria were incubated in a 37°C in a roller at 60 rpm. Samples were removed and visualized on a glass slide at the indicated times using a Zeiss LSM 510 confocal laser-scanning microscope. Image series were processed using Volocity (Improvision).

## Results

### Depletion Aggregation can Actuate Cell-Cell Adhesion by Exopolysaccharides.

*P. aeruginosa* encodes three exopolysaccharides: Pel is a cationic polymer composed of partially acetylated N-acetylgalactosamine and N-acetylglucosamine (Jennings et al., 2015), Psl is a neutral polymer containing glucose, mannose, and rhamnose (Byrd et al., 2009), and alginate is a negatively-charged polymer composed of mannuronic and guluronic acid (Pedersen et al., 1992; Gibson et al., 2003).

We first tested wild-type *P. aeruginosa* PAO1 that encodes all three exopolysaccharides (Colvin et al., 2012; Wiens et al., 2014). As seen previously, wild-type *P. aeruginosa* exposed to the model polymer PEG (35 kDa) rapidly aggregated *via* the depletion mechanism, but disaggregated when polymers were diluted by adding PBS (**Figure 2A**). Adding PEG did not disperse aggregates, implicating polymer dilution rather than physical disruption in disaggregation (**Figure S1**). Notably, wild-type *P. aeruginosa* aggregates held together by PEG exposure for as long as 18 hours disaggregated upon polymer dilution with PBS (**Figure 2A** and **Figure S2**). Reversibility with dilution is a hallmark of depletion aggregation, as it is driven by a reduction in crowding effects of environmental polymers. Thus, in the conditions tested, wild-type *P. aeruginosa* did not activate bacterially-driven adhesive mechanisms to maintain aggregation.

Expression of exopolysaccharides is a key step in surface adherence and aggregation in surface-associated biofilms (Mann and Wozniak, 2012), so we reasoned that strains overproducing exopolysaccharides might remain aggregated after polymer dilution. To test this, we aggregated *P. aeruginosa* PAO1 overproducing alginate, Pel, or Psl, and investigated whether PBS dilution caused dispersal. Alginate overproduction was achieved *via* a mutation in an anti-sigma factor gene regulating alginate (PAO1 *mucA22*), and Pel or Psl overproduction was achieved using Pel or Psl genes on an inducible promoter (PAO1 P_BAD_-Psl and PAO1 P_BAD_-Pel).

After 18 hours of depletion aggregation, wild-type PAO1 and PAO1 overproducing alginate (PAO1 *mucA22*) readily dispersed after polymer dilution (i.e. PBS addition) (**Figures 2A, B**) whereas the strains over-expressing Pel and Psl did not (**Figures 2C, D** and **Figure S2**). These findings indicate that cells aggregated by the depletion mechanism that have Pel and Psl expression induced can remain aggregated after depletion promoting-conditions are reversed.

To determine if the differential effects of alginate verses Pel and Psl on aggregate stability were generalizable to strains other than PAO1, we studied *P. aeruginosa* clinical isolates taken from people with CF. CF strains can evolve exopolysaccharide over-expression phenotypes (Smith et al., 2006). Pel or Psl overexpression is known to produce a rugose small-colony morphology (Starkey et al., 2009) whereas strains that over-produce alginate are mucoid (Pedersen et al., 1992).

All 10 P*. aeruginosa* CF clinical isolates tested that had a rugose colony morphology formed dilution-resistant aggregates in PEG (**Figure 2E** and **Table 2**), whereas all (9/9) alginate-overproducing clinical isolates (i.e. mucoid strains) had a reversible aggregation phenotype (**Figure 2F** and **Table 2**). These results with exopolysaccharide-overproducing clinical isolates are consistent with findings using engineered PAO1 strains (see above) and suggest that induced expression of Pel and Psl, but not alginate, enable aggregates formed by the depletion mechanism to remain intact after depletion-promoting conditions are reversed. Different chemical compositions or physical properties of bacterial exopolysaccharides such as charge may explain these differences.

**Table 2:** *P. aeruginosa* morphology and aggregate reversibility phenotypes.

Strain	Morphology	Reversible aggregation?
PAO1	Non-mucoid	Yes
PAO1 Δ*wspF/pslD*; pBAD::Pel	Non-mucoid	No
PAO1 Δ*wspF/pelF*; pBAD::Psl	Non-mucoid	No
PDO300 mucA22	Mucoid	Yes
PAO1 Δ*mucA*	Mucoid	Yes
Clinical Isolate 2-6.3	Mucoid	Yes
Clinical Isolate 29-14	Mucoid	Yes
Clinical Isolate 7-15.4	Mucoid	Yes
Clinical Isolate 9-19.6A	Mucoid	Yes
Clinical Isolate W1	Mucoid	Yes
Clinical Isolate W2	Mucoid	Yes
Clinical Isolate W3	Mucoid	Yes
Clinical Isolate W4	Mucoid	Yes
Clinical Isolate W5	Mucoid	Yes
Clinical Isolate 27-6.4	Rugose	No
Clinical Isolate 28-17.9	Rugose	No
Clinical Isolates 29-5.6	Rugose	No
Clinical Isolate 14-4.2	Rugose	No
Clinical Isolate 17-6.6	Rugose	No
Clinical Isolate S1	Rugose	No
Clinical Isolate S2	Rugose	No
Clinical Isolate S3	Rugose	No
Clinical Isolate S4	Rugose	No
Clinical Isolate S5	Rugose	No

Previous work shows that non-adsorbing biological polymers can produce depletion aggregation of bacteria like PEG does (Dorken et al., 2012; Secor et al., 2018). However, polymers found at infection sites can also induce biological responses in bacteria that have consequences for aggregation whereas PEG is considered to be relatively inert (Banerjee et al., 2012). For example, exposure to mucin can induce the expression of *P. aeruginosa* genes important in infection pathogenesis (Lory et al., 1996; Wang et al., 1996). Our previous work indicates that depletion aggregates formed *in vitro* by exposing *P. aeruginosa* to mixtures of mucin and DNA are comparable in size to aggregates formed by PEG (Secor et al., 2018). These observations led us to investigate whether depletion aggregates induced by biological polymers exhibit dispersal after polymer dilution, like aggregates induced by PEG.

To test this, we induced depletion aggregation using a mixture of mucin and DNA, which are major polymers in lung secretions (i.e. sputum) from people with CF. In these experiments we used concentrations found similar to those *in vivo* (mucin at 4% w/vol and DNA at 2 mg/ml) (Secor et al., 2018), and fluorescently-tagged bacterial strains because mucin/DNA mixtures are opaque (PEG is transparent), and assayed several hundred aggregates per condition. Similar to PEG, mucin and DNA mixtures aggregated wild-type PAO1 and PBS addition 15 minutes later caused aggregate dispersal (**Figure 3A** **;** **Figure S3**). However, when we extended the period of polymer aggregation to 120 minutes, wild-type PAO1 that had been aggregated in mucin/DNA mixtures remained intact after PBS dilution and mixing by vortexing (**Figure 3B** **;** **Figure S3**), whereas those that had been aggregated in PEG dispersed (**Figure 3B**). These results suggest that the aggregates that survive dilution by PBS and mixing are stable and in a steady state.

Our finding that induced expression of Pel and Psl makes depletion aggregates dilution-resistant led us to investigate whether self-produced exopolysaccharides mediated the dilution-resistant phenotype of aggregates induced by mucin and DNA. We tested this using PAO1 in which biosynthetic genes of all three exopolysaccharides had been inactivated (PAO1Δ*pelA/pslBCD/algD*) and found that the mutant lacking exopolysaccharides genes dispersed upon dilution with PBS regardless of whether aggregation was induced by PEG or the mucin/DNA mixture (**Figures 3C, D**). Collectively, these results suggest that depletion aggregation can actuate cell-cell adhesion by some *P. aeruginosa* exopolysaccharides, and that depletion aggregation by polymers present at infection sites can initiate the formation of aggregates that remain intact after depletion-mediating conditions are reversed provided exopolysaccharide genes are intact.

### Cell Shape Is Associated With Species Distribution in Depletion Aggregates

Theory predicts that bacteria aggregated by the depletion mechanism will be arranged to minimize the amount of volume occupied (Poon, 2002), as efficient packing will increase the space available for polymers and the concomitant entropy gains. This effect should cause bacteria with similar shapes to be arranged together, and bacteria with different shapes to separate, unless other external forces or bacterial activity intervene. To test this hypothesis, we mixed *P. aeruginosa*, *Burkholderia cenocepacia* (rod)*, Escherichia coli* (rod), and *Staphylococcus aureus* (a coccus) bearing different florescent labels in various combinations in PEG and examined species distribution by microscopy.

**Figure 4:**
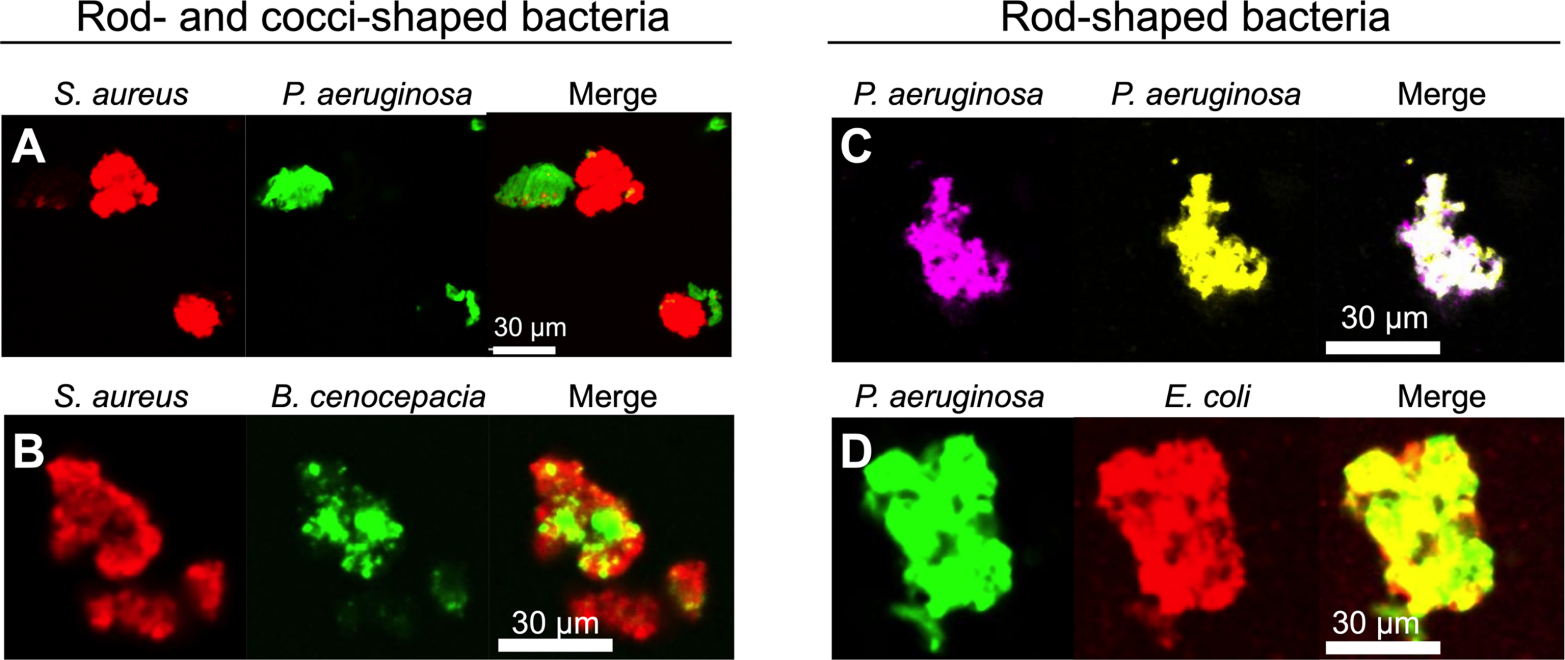
Depletion aggregation spontaneously segregates bacteria with different cell shapes. Equal numbers of the indicated species (10^9^ CFU/ml each) were mixed prior to the addition of PEG 35 kDa to induce depletion aggregation. After 18 hours, aggregates were imaged by fluorescent microscopy. Representative images of depletion aggregates composed of **(A)** *P. aeruginosa* expressing GFP and *S. aureus* expressing mCherry, **(B)** *S. aureus* expressing mCherry and *B. cenocepacia* expressing GFP, **(C)** *P. aeruginosa* expressing either YFP or CFP, or **(D)** *P. aeruginosa* expressing GFP and *E. coli* expressing mCherry are shown.

Polymer-mediated depletion aggregation caused cocci-shaped species (*S. aureus*) to segregate from rods (*P. aeruginosa* and *B. cenocepacia*). Two patterns of segregation were observed. In some cases, entire aggregates appeared to be composed of a single species (i.e either rods or cocci) without an appreciable presence of the differently shaped species (**Figure 4A**). In other cases, sections of mixed-species aggregates were composed primarily of either the rod or cocci-shaped species, as shown with *S. aureus* and *B. cenocepacia* (**Figure 4B**). Similar results were seen using mixtures of formalin-killed *P. aeruginosa* and *S. aureus*, and with *P. aeruginosa* mixed with 2 µm diameter spherical beads similarly sized as *S. aureus* (**Figures S4A, B**). Thus, bacterial activity is not required for species segregation under the conditions tested.

In contrast, depletion aggregation caused bacteria with similar cell shapes (i.e. differentially labeled *P. aeruginosa* with *P. aeruginosa, or P. aeruginosa* with *E. coli*) to intermix (**Figures 4C, D**). These experiments, along with previous work using inert particles (Adams et al., 1998), show that physical forces mediating depletion aggregation cause like-shaped bacteria to intermix, and differently shaped bacteria to separate. The physical arrangement of bacterial species in aggregates can affect competitive and cooperative interactions (see below).

### Depletion Aggregation Promotes Antimicrobial Tolerance in *S. aureus*.

Our finding that depletion aggregation can determine the physical arrangement of species within aggregates led us to investigate its effects on interspecies interactions. *P. aeruginosa* and *S. aureus* are often co-isolated from CF airways (Harrison, 2007; Hauser et al., 2011) and wounds (Kirketerp-Moller et al., 2008; DeLeon et al., 2014) for long durations. However, in laboratory co-cultures, *P. aeruginosa* rapidly inhibits *S. aureus* by quorum-regulated antimicrobials such as rhamnolipids, hydrogen cyanide, phenazines, quinolones, and others (Mavrodi et al., 2001; Deziel et al., 2004; Mashburn et al., 2005; Palmer et al., 2005; Schuster and Greenberg, 2006). Because aggregation can increase antimicrobial tolerance (Haaber et al., 2012; Staudinger et al., 2014), we hypothesized that depletion aggregation could enhance the ability of *S. aureus* to co-exist with *P. aeruginosa*.

**Figure 5:**
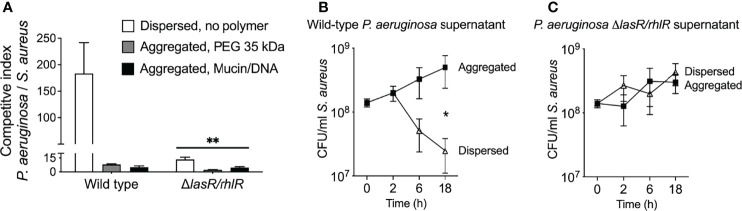
Depletion aggregation increases *S. aureus* tolerance to quorum-regulated antimicrobials secreted by *P. aeruginosa*. **(A)** Equal numbers (10^7^ CFUs) of *S. aureus* and *P. aeruginosa* (wild-type PAO1 or Δ*lasR/rhlR*) were cocultured in LB supplemented with 30% w/vol PEG 35 kDa or mucin (4%w/vol) and DNA (2 mg/ml), where indicated. After 18-h, viable bacteria were enumerated by serial dilution and plating and plotting the competitive index (change [final/initial] in *P. aeruginosa* vs. *S. aureus* CFUs). Results are the mean ± SD, N = 3 for each condition; **p<0.01 relative to wild type, Student’s *t*-test. **(B, C)** *S. aureus* (10^8^ CFU/ml) was added to filter sterilized supernatants collected from wild-type or Δ*lasR/rhlR P. aeruginosa* overnight cultures supplemented with 30% w/vol PEG 35 kDa where indicated. Viable *S. aureus* was enumerated by serial dilution and plating at the indicated times. Results are the mean ± SD, N = 3 for each condition and timepoint; *p < 0.02, Student’s *t*-test.

Similar to previous studies (Mavrodi et al., 2001; Deziel et al., 2004; Mashburn et al., 2005; Palmer et al., 2005; Schuster and Greenberg, 2006), we found that wild-type *P. aeruginosa* severely inhibited *S. aureus* in non-aggregated broth co-cultures (**Figure 5A**), and inhibition was diminished if quorum sensing was genetically inactivated (i.e. using Δ*lasI/rhlI* PAO1) (**Figure 5A**, compare white bars). However, in co-cultures where PEG or mucin/DNA was used to induce depletion aggregation, the competitive index of wild-type *P. aeruginosa* over *S. aureus* was reduced by greater than 10-fold (**Figure 5A**, gray and black bars).

Previous work indicating that depletion aggregation caused marked tolerance of *P. aeruginosa* to pharmaceutical antibiotics (Secor et al., 2018) led us to investigate whether depletion aggregation could cause *S. aureus* to become insensitive to antimicrobials produced by *P. aeruginosa*. We tested this by exposing dispersed and depletion-aggregated *S. aureus* to filter-sterilized *P. aeruginosa* planktonic culture supernatants. Supernatants from wild-type *P. aeruginosa* killed ~10-fold more dispersed *S. aureus* than aggregated *S. aureus* (**Figure 5B**), whereas supernatants from *P. aeruginosa* Δ*lasI/rhlI* did not kill dispersed or aggregated *S. aureus* (**Figure 5C**). Control experiments indicate that PEG did not diminish the antimicrobial activity of wild-type *P. aeruginosa* supernatants (**Figure S5**). These results suggest that depletion aggregation may promote co-existence of *P. aeruginosa* and *S. aureus* by enhancing *S. aureus* tolerance to quorum-regulated antimicrobials secreted by *P. aeruginosa*. It is also possible that decreased production of antimicrobial factors by aggregated *P. aeruginosa* contributes to species co-existence in aggregates.

### Depletion Aggregation Promotes Contact-Dependent Bacterial Antagonism

In addition to secreted factors, *P. aeruginosa* and other bacteria also possess competitive mechanisms that depend upon direct cell-to-cell contact. One mechanism is type VI secretion (TSS) in which a needle-like apparatus delivers toxins and effectors into neighboring cells (Mougous et al., 2006). Our finding that depletion aggregation causes like-shaped bacterial cells to intermix in aggregates led us to hypothesize that depletion aggregation could promote TSS-mediated bacterial antagonism.

**Figure 6:**
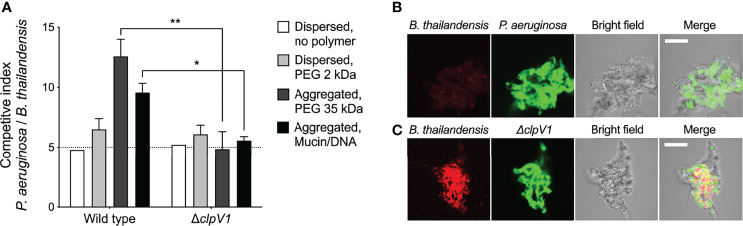
Depletion aggregation promotes contact-dependent bacterial competition. **(A)** The outcome of competitions between *B. thailandensis* and either wild-type or Δ*clpV1 P. aeruginosa* are shown. Initial cultures contained 1x10^8^ CFU/ml *P. aeruginosa* and 2x10^7^ CFU/ml *B. thailandensis.* Results are after 24-h of co-culture in the indicated conditions and are the mean ± SD, N = 3 for each condition; **p < 0.01, Student’s *t*-test. **(B, C)** Confocal microscopy was used to visualize depletion aggregates after 24-h of co-culture with 30% PEG 35 kDa. Representative 10µm thick sections through the aggregates are shown with **(B)**. thailandensis strains in red and *P. aeruginosa* in green. Scale bar, 30 µm. *P < 0.05.

To test this, we mixed *P. aeruginosa* (which is capable of TSS antagonism) with *Burkholderia thailandensis*, a TSS-susceptible rod-shaped Gram-negative bacterium (LeRoux et al., 2012) at a 5:1 ratio following previously established protocols (LeRoux et al., 2015). In dispersed conditions, no *P. aeruginosa*-*B. thailandensis* antagonism was apparent over 24 hours, as the ratio of *P. aeruginosa* to *B. thailandensis* remained unchanged at 5:1 (**Figure 6A**). In contrast, *P. aeruginosa* outcompeted *B. thailandensis* in depletion aggregates as measured by viable counts (**Figure 6A**) and visually assessing differentially-labeled species (**Figure 6B**). Notably the aggregation-induced competitive advantage of *P. aeruginosa* was eliminated by genetically inactivating TSS [i.e. PAO1 Δ*clpV1* (**Figure 6C**)]. The reduced fluorescent signal of *B. thailandensis* could be due to cell death or inhibition. Taken together, these results demonstrate that depletion aggregation can facilitate contact-dependent mechanisms of bacterial antagonism.

## Discussion

Pathogens causing chronic infection like those in CF airways and wounds are generally found in aggregates suspended in polymer-rich secretions (Costerton et al., 1995; Singh et al., 2000; Worlitzsch et al., 2002; Kirketerp-Moller et al., 2008; Bjarnsholt et al., 2009; Fazli et al., 2009; Bjarnsholt et al., 2013; DePas et al., 2016; Stacy et al., 2016; Sonderholm et al., 2017; Bay et al., 2018; Kim et al., 2020; Jennings et al., 2021). Our previous work shows that physical forces produced by polymers found at infection sites can cause bacteria to form suspended aggregates by the depletion mechanism, and depletion aggregation produces antimicrobial tolerance phenotypes (Secor et al., 2018). In this study we found that depletion aggregation can (i) actuate bridging interactions mediated by two of *P. aeruginosa*’s self-produced biofilm polysaccharides, (ii) cause bacteria with similar shapes to intermix and bacteria with different shapes to segregate, and (iii) can influence the outcome of bacterial competition mediated by secreted factors and cell-to-cell contact.

Surface attachment induces biofilm formation *via* several mechanisms. Sensing and adhering to surfaces induces physiological responses important in biofilm growth, and attachment keeps nascent biofilm-forming cells from dispersing (from random movement or fluid flows) giving self-produced matrix material the opportunity to bind cells together (O'Toole et al., 2000). Our work raises the possibility that the depletion mechanism can serve similar functions for suspended aggregates as attachment surfaces serve for biofilms. For example, previously we found that like surface attachment (Wood and Ohman, 2009), depletion aggregation can induce stress responses in *P. aeruginosa* that mediate antibiotic tolerance (Secor et al., 2018). Our current experiments show that depletion aggregation also brings suspended cells together and can promote cell-cell adhesion by self-produced exopolysaccharides.

One important caveat is that when PEG was used to induce depletion aggregation, exopolysaccharide overexpression was required as wild-type *P. aeruginosa* PAO1 capable of producing exopolysaccharides did not produce aggregates that remained intact after polymer dilution, even after long exposures to PEG. However, when mucin/DNA mixtures were used to induce depletion aggregation, wild-type *P. aeruginosa* did exhibit durable aggregation that was resistant to dispersal by dilution. Notably, *P. aeruginosa* strains constitutively expressing exopolysaccharides can be isolated from infected CF subjects (Starkey et al., 2009), and it is possible that *in vivo* conditions (such mucin and DNA in CF airway secretions) could induce exopolysaccharide expression to produce durable aggregation.

**Figure 7:**
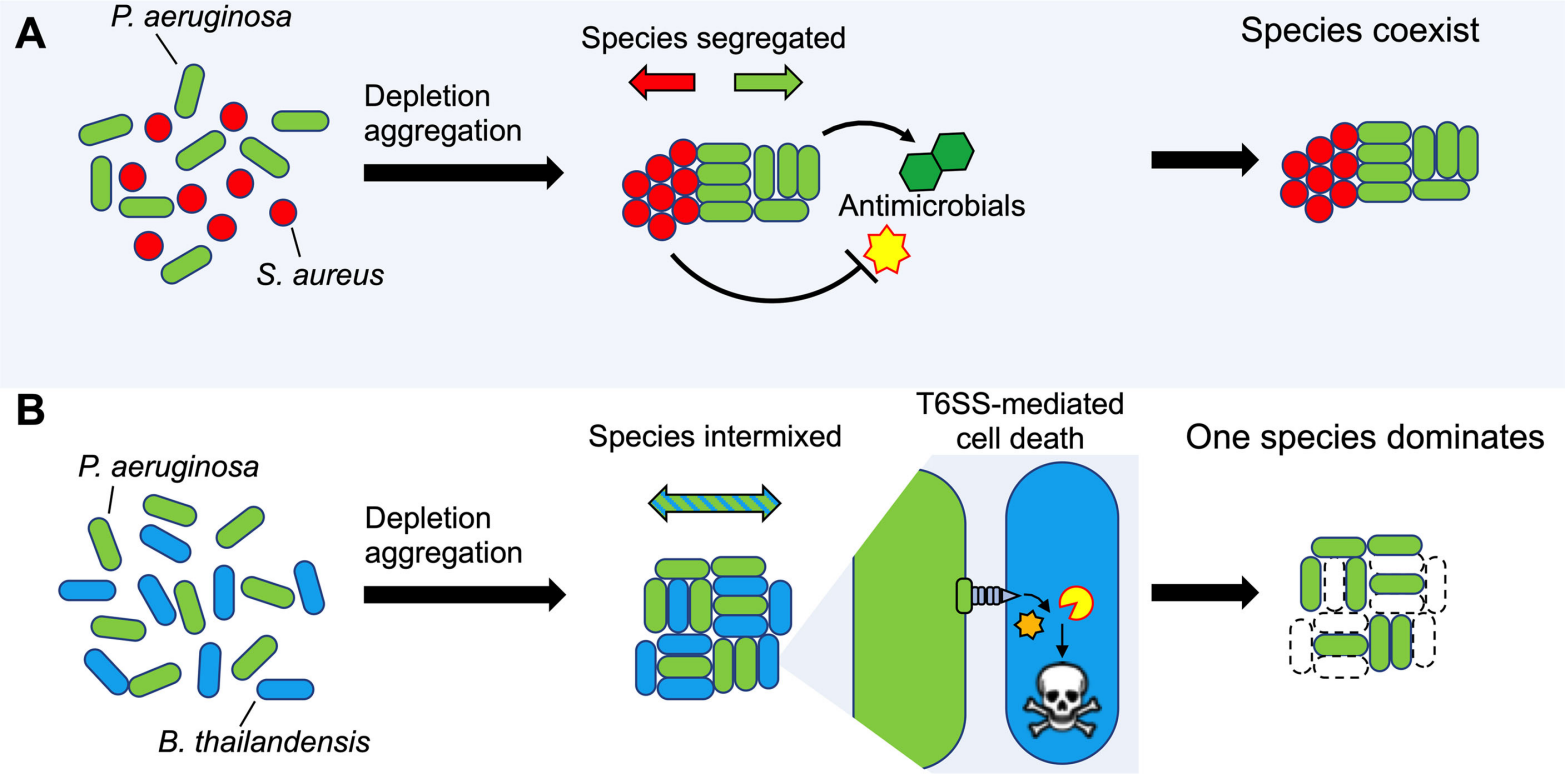
Model depicting how depletion aggregation affects bacterial competition and species distribution in aggregates. **(A)** Depletion aggregation causes bacteria with different cell shapes to spontaneously segregate. When *P. aeruginosa* and *S. aureus* were co-cultured under conditions promoting depletion aggregation, *S. aureus* aggregates tolerated antimicrobials secreted by *P. aeruginosa*, promoting species coexistence. **(B)** When two rod-shaped species such as *P. aeruginosa* and *B. thailandensis* are aggregated by the depletion mechanism, species segregation is not observed and contact-dependent TSS-mediated killing is promoted, allowing *P. aeruginosa* to dominate.

Our findings also have implications for interspecies interactions that may occur in infections. The experiments showing that depletion aggregation increases tolerance of *S. aureus* to antimicrobials produced by *P. aeruginosa* (**Figure 7A**) could help explain how *P. aeruginosa* and *S. aureus* can co-exist in chronic infections like wounds and CF lungs (Fischer et al., 2021), but are difficult to maintain in liquid co-cultures in the laboratory. While the underlying mechanism remains to be characterized, our previous work showing that depletion aggregation induces the SOS stress response in *P. aeruginosa* (Secor et al., 2018) raises the possibility that a similar phenomenon operates in *S. aureus* (Anderson et al., 2006; Gardete et al., 2006). If general stresses were induced, aggregated *S. aureus* may exhibit tolerance to other environmental stresses including antibiotics. It is also possible that aggregated *P. aeruginosa* produce less antimicrobials compared to planktonic cultures, and this may also contribute to co-existence.

The species could have wide ranging effects. One consequence we demonstrated is enhanced efficacy of TSS-mediated inhibition of rod shaped *Burkholderia* sp. by rod-shaped *P. aeruginosa*, as TSS is dependent upon species intermixing and prolonged cell-to-cell contact (**Figure 7B**). Such interactions could contribute to the ability of *P. aeruginosa* to dominate other rod-shaped CF pathogens such as *Haemophilus influenzae* and *Stenotrophomonas maltophilia* (Harrison, 2007; Coutinho et al., 2008; Hauser et al., 2011; Schwab et al., 2014; Jorth et al., 2015). Depletion aggregation could likewise affect other close-range mechanisms that depend on contact or have short diffusion distances (like oxidants), depending on whether species are of similar or dissimilar shapes. In addition, in settings where depletion aggregation is maintained for long durations (i.e. polymers are continuously present), the effects of depletion aggregation on species arrangement could shape co-evolutionary trajectories of species, as the within-aggregate arrangement of cells likely affects selection, competition, and cell migration.

In addition to bacteria that cause infection, depletion aggregation may also affect interactions between commensal bacterial species. Recent work examining TSS in *Vibrio fischeri* as it transitions from life in an aquatic environment to colonization of the squid host reveals that viscous, polymer-rich host conditions promote both *V*. *fischeri* aggregation and TSS activation, which increases fitness against competing bacteria during initial stages of host colonization (Speare et al., 2020). The addition of the polymer polyvinylpyrrolidone (PVP) to *V. fischeri* cultures *in vitro* was used to increase the viscosity of the growth medium to experimentally validate TSS activation. Notably, PVP also induces depletion aggregation of inert bacteria-sized colloids (McFarlane et al., 2010). It would be interesting to dissect the contributions of viscosity and depletion aggregation on bacterial aggregate assembly and interspecies competition in both pathogenic and commensal settings.

Our study had several limitations. In some experiments we used a non-biological polymer (PEG) at a specific concentration (30% w/vol) with a defined molecular weight (PEG 35 kDa) to induce depletion aggregation. Use of a defined polymer limited variability and the transparency of PEG enhanced microscopy. We confirmed key findings with biological polymers (mucin and DNA), but for feasibility reasons we used porcine gastric mucin and salmon sperm DNA which are supplied as lyophilized powders. Thus, it is possible that biological polymers could produce different results *in vivo*. We think this is unlikely because polymers with disparate chemical properties produce depletion aggregates with similar morphologies and tolerance phenotypes (Secor et al., 2018).

We also recognize that varying polymer size and molecular weight will affect the strength of the aggregating force by changing osmotic pressure. Furthermore, the presence of multivalent cations could introduce polymer-polymer bridging interactions that would affect results, and these variables were not examined here. We also only explored intermixing in rod-shaped bacteria; it is likely that intermixing also occurs in populations of cocci-shaped bacterial species, which would be consistent with experiments using inert colloidal particles (Li et al., 2020). An additional limitation was that our experiments used laboratory strains and a handful of *P. aeruginosa* clinical isolates. Clinical isolates with different biological characteristics could affect depletion-mediated bacteria-bacteria interactions. For example, recent work demonstrates that LPS O-antigen modifications in *P. aeruginosa* change cell surface hydrophobicity, which may disrupt the tightly-packed and ordered cell arrangements characteristic of depletion aggregates (Azimi et al., 2021). Other surface modifications that affect surface charge could also affect depletion-mediated bacteria-bacteria or bacteria-polymer interactions. Finally, we did not explore the contributions of pili, flagella, adhesins, or exopolysaccharide binding proteins such as CdrA (Borlee et al., 2010), which could be important in stabilizing bacterial aggregates formed by PAO1 or by clinical *P. aeruginosa* isolates.

Much research in model systems has been devoted to understanding bacterial sensing and signaling pathways, purpose-evolved genetic programs, and cooperation behaviors that shape bacterial phenotypes important in chronic infections. The data presented here show that physical forces inherent to polymer-rich environments can have marked effects on complex bacterial behaviors including aggregation, stress survival, and interspecies competition. New strategies to manipulate pathogenesis phenotypes will require understanding the relative contributions of bacterially-driven processes and mechanisms caused by physical forces in the environment.

## Data Availability Statement

The original contributions presented in the study are included in the article/**Supplementary Material**. Further inquiries can be directed to the corresponding author.

## Author Contributions

All authors listed have made a substantial, direct, and intellectual contribution to the work and approved it for publication.

## Funding

NIH grants K22AI125282, R01AI138981, and P30GM140963 to PRS; R01HL141098-01A1 to PKS. Isolates were provided by the Clinical Core of UW’s CF Foundation sponsored Research Development Program (SINGH19R0).

## Conflict of Interest

The authors declare that the research was conducted in the absence of any commercial or financial relationships that could be construed as a potential conflict of interest.

## Publisher’s Note

All claims expressed in this article are solely those of the authors and do not necessarily represent those of their affiliated organizations, or those of the publisher, the editors and the reviewers. Any product that may be evaluated in this article, or claim that may be made by its manufacturer, is not guaranteed or endorsed by the publisher.

## Supplementary Material

Supplementary Figure 1 Depletion aggregation was induced with 30% w/vol PEG 35 kDa for 18 hours. *P. aeruginosa* PAO1 depletion aggregates were then diluted 10X with additional PEG 35 kDa. Scale bar 40 µm.

Supplementary Figure 2 Depletion aggregate dispersal phenotypes of *P. aeruginosa* PAO1 and PAO1 pBAD::Pel. Aggregate dispersal of wild-type and pBAD::Pel strains was measured. Depletion aggregation was induced with 30% w/vol PEG 35 kDa for 18 hours. Depletion aggregates were then diluted 10X with PBS and representative images were acquired pre-dilution and 10- and 60-seconds post-dilution.

Supplementary Figure 3 Depletion aggregate dispersal phenotypes of *P. aeruginosa* in mixtures of mucin and DNA. Aggregate dispersal of fluorescent *P. aeruginosa* PAO1 was measured. Depletion aggregation was induced with 4% w/vol mucin and 2 mg/ml DNA for 15 or 120 minutes. Depletion aggregates were then diluted 10X with PBS and images were acquired immediately pre- and immediately post-dilution. Representative images are shown.

Supplementary Figure 4 Depletion aggregation operates on dead cells and inert latex beads. **(A, B)** Depletion aggregation was induced with 30% w/vol PEG 35 kDa using combinations of the indicated species of dead formalin-fixed bacteria. Fluorescent microscopy was used to image aggregates after 18-h of growth. Bar, 30 µm. **(C)** *P. aeruginosa* (white arrows) and fluorescent spherical latex beads (2 µm diameter, black arrows) were aggregated using 30% w/vol PEG 35 kDa for 18-h and imaged using fluorescent and brightfield microscopy. Bar, 30 µm.

Supplementary Figure 5 PEG does not inactivate antimicrobials present in *P. aeruginosa* supernatants. One possible explanation for the reduced killing of aggregated *S. aureus* (see ) was that PEG somehow inactivated antimicrobials present in wild-type *P. aeruginosa* supernatants. To address this possibility, we used a lower molecular weight PEG (PEG 2 kDa). As polymer molecular weight decreases, the polymer concentration required to induce depletion aggregation of a given number of cells increases. Thus, PEG 2 kDa does not promote depletion aggregation at 30% w/vol. Dissolving PEG 2 kDa into wild-type *P. aeruginosa* supernatants did not affect *S. aureus* inhibition in supernatants collected from **(A)** wild-type or **(B)** Δ*lasR/rhlR* overnight cultures compared to polymer-free controls, indicating that PEG did not inactivate antimicrobials present in *P. aeruginosa* supernatants.

## References

[B1] Adams M. Dogic Z. Keller S. L. Fraden S. (1998). Entropically Driven Microphase Transitions in Mixtures of Colloidal Rods and Spheres. Nature 393, 349–352. doi: 10.1038/30700

[B2] Alhede M. Kragh K. N. Qvortrup K. Allesen-Holm M. van Gennip M. Christensen L. D.. (2011). Phenotypes of Non-Attached Pseudomonas Aeruginosa Aggregates Resemble Surface Attached Biofilm. PloS One 6, e27943. doi: 10.1371/journal.pone.0027943 22132176 PMC3221681

[B3] Anderson K. L. Roberts C. Disz T. Vonstein V. Hwang K. Overbeek R.. (2006). Characterization of the Staphylococcus Aureus Heat Shock, Cold Shock, Stringent, and SOS Responses and Their Effects on Log-Phase mRNA Turnover. J. Bacteriol 188, 6739–6756. doi: 10.1128/JB.00609-06 16980476 PMC1595530

[B4] Asakura S. Oosawa F. (1958). Interaction Between Particles Suspended in Solutions of Macromolecules. J. Polymer Sci. 33, 183–192. doi: 10.1002/pol.1958.1203312618

[B5] Azimi S. Thomas J. Cleland S. E. Curtis J. E. Goldberg J. B. Diggle S. P. (2021). O-Specific Antigen-Dependent Surface Hydrophobicity Mediates Aggregate Assembly Type in Pseudomonas Aeruginosa. mBio 12 (4), e0086021. doi: 10.1128/mBio.00860-21 34372703 PMC8406328

[B6] Banerjee S. S. Aher N. Patil R. Khandare J. (2012). Poly(ethylene Glycol)-Prodrug Conjugates: Concept, Design, and Applications. J. Drug Delivery 2012, 103973. doi: 10.1155/2012/103973 PMC3356704 22645686

[B7] Bay L. Kragh K. N. Eickhardt S. R. Poulsen S. S. Gjerdrum L. M. R. Ghathian K.. (2018). Bacterial Aggregates Establish at the Edges of Acute Epidermal Wounds. Adv. Wound Care (New Rochelle) 7, 105–113. doi: 10.1089/wound.2017.0770 29675336 PMC5905854

[B8] Bjarnsholt T. Alhede M. Eickhardt-Sorensen S. R. Moser C. Kuhl M. Jensen P. O.. (2013). The *In Vivo* Biofilm. Trends Microbiol. 21, 466–474. doi: 10.1016/j.tim.2013.06.002 23827084

[B9] Bjarnsholt T. Jensen P. O. Fiandaca M. J. Pedersen J. Hansen C. R. Andersen C. B.. (2009). Pseudomonas Aeruginosa Biofilms in the Respiratory Tract of Cystic Fibrosis Patients. Pediatr. Pulmonol. 44, 547–558. doi: 10.1002/ppul.21011 19418571

[B10] Blom J. F. Zimmermann Y. S. Ammann T. Pernthaler J. (2010). Scent of Danger: Floc Formation by a Freshwater Bacterium Is Induced by Supernatants From a Predator-Prey Coculture. Appl. Environ. Microbiol. 76, 6156–6163. doi: 10.1128/AEM.01455-10 20656874 PMC2937497

[B11] Borlee B. R. Goldman A. D. Murakami K. Samudrala R. Wozniak D. J. Parsek M. R. (2010). Pseudomonas Aeruginosa Uses a Cyclic-Di-GMP-Regulated Adhesin to Reinforce the Biofilm Extracellular Matrix. Mol. Microbiol. 75, 827–842. doi: 10.1111/j.1365-2958.2009.06991.x 20088866 PMC2847200

[B12] Byrd M. S. Sadovskaya I. Vinogradov E. Lu H. Sprinkle A. B. Richardson S. H.. (2009). Genetic and Biochemical Analyses of the Pseudomonas Aeruginosa Psl Exopolysaccharide Reveal Overlapping Roles for Polysaccharide Synthesis Enzymes in Psl and LPS Production. Mol. Microbiol. 73, 622–638. doi: 10.1111/j.1365-2958.2009.06795.x 19659934 PMC4409829

[B13] Choi K. H. Schweizer H. P. (2006). Mini-Tn7 Insertion in Bacteria With Single Atttn7 Sites: Example Pseudomonas Aeruginosa. Nat. Protoc. 1, 153–161. doi: 10.1038/nprot.2006.24 17406227

[B14] Clark R. A. F. (1996). The Molecular and Cellular Biology of Wound Repair. 2nd ed (New York: Plenum Press).

[B15] Colvin K. M. Alnabelseya N. Baker P. Whitney J. C. Howell P. L. Parsek M. R. (2013). PelA Deacetylase Activity Iss Required for Pel Polysaccharide Synthesis in Pseudomonas Aeruginosa. J. Bacteriol. 195, 2329–2339. doi: 10.1128/JB.02150-12 23504011 PMC3650530

[B16] Colvin K. M. Irie Y. Tart C. S. Urbano R. Whitney J. C. Ryder C.. (2012). The Pel and Psl Polysaccharides Provide Pseudomonas Aeruginosa Structural Redundancy Within the Biofilm Matrix. Environ. Microbiol. 14, 1913–1928. doi: 10.1111/j.1462-2920.2011.02657.x 22176658 PMC3840794

[B17] Costerton J. W. Lewandowski Z. Caldwell D. E. Korber D. R. Lappin-Scott H. M. (1995). Microbial Biofilms. Annu. Rev. Microbiol. 49, 711–745. doi: 10.1146/annurev.mi.49.100195.003431 8561477

[B18] Coutinho H. D. Falcao-Silva V. S. Goncalves G. F. (2008). Pulmonary Bacterial Pathogens in Cystic Fibrosis Patients and Antibiotic Therapy: A Tool for the Health Workers. Int. Arch. Med. 1, 24. doi: 10.1186/1755-7682-1-24 18992146 PMC2586015

[B19] Davey M. E. O'Toole G. A. (2000). Microbial Biofilms: From Ecology to Molecular Genetics. Microbiol. Mol. Biol. Rev. 64, 847–867. doi: 10.1128/MMBR.64.4.847-867.2000 11104821 PMC99016

[B20] DeLeon S. Clinton A. Fowler H. Everett J. Horswill A. R. Rumbaugh K. P. (2014). Synergistic Interactions of Pseudomonas Aeruginosa and Staphylococcus Aureus in an *In Vitro* Wound Model. Infect. Immun. 82, 4718–4728. doi: 10.1128/IAI.02198-14 25156721 PMC4249327

[B21] DePas W. H. Starwalt-Lee R. Van Sambeek L. Kumar S. R. Gradinaru V. Newman D. K. (2016). Exposing the Three-Dimensional Biogeography and Metabolic States of Pathogens in Cystic Fibrosis Sputum *via* Hydrogel Embedding, Clearing, and rRNA Labeling. mBio 7, e00796–e00716. doi: 10.1128/mBio.00796-16 27677788 PMC5040109

[B22] Deziel E. Lepine F. Milot S. He J. Mindrinos M. N. Tompkins R. G.. (2004). Analysis of Pseudomonas Aeruginosa 4-Hydroxy-2-Alkylquinolines (HAQs) Reveals a Role for 4-Hydroxy-2-Heptylquinoline in Cell-to-Cell Communication. Proc. Natl. Acad. Sci. U. S. A. 101, 1339–1344. doi: 10.1073/pnas.0307694100 14739337 PMC337054

[B23] Dorken G. Ferguson G. P. French C. E. Poon W. C. K. (2012). Aggregation by Depletion Attraction in Cultures of Bacteria Producing Exopolysaccharide. J. R. Soc. Interface 9, 3490–3502. doi: 10.1098/rsif.2012.0498 22896568 PMC3481587

[B24] Fazli M. Bjarnsholt T. Kirketerp-Moller K. Jorgensen B. Andersen A. S. Krogfelt K. A.. (2009). Nonrandom Distribution of Pseudomonas Aeruginosa and Staphylococcus Aureus in Chronic Wounds. J. Clin. Microbiol. 47, 4084–4089. doi: 10.1128/JCM.01395-09 19812273 PMC2786634

[B25] Fischer A. J. Singh S. B. LaMarche M. M. Maakestad L. J. Kienenberger Z. E. Pena T. A.. (2021). Sustained Coinfections With Staphylococcus Aureus and Pseudomonas Aeruginosa in Cystic Fibrosis. Am. J. Respir. Crit. Care Med. 203, 328–338. doi: 10.1164/rccm.202004-1322OC 32750253 PMC7874317

[B26] Gardete S. Wu S. W. Gill S. Tomasz A. (2006). Role of VraSR in Antibiotic Resistance and Antibiotic-Induced Stress Response in Staphylococcus Aureus. Antimicrob. Agents Chemother. 50, 3424–3434. doi: 10.1128/AAC.00356-06 17005825 PMC1610096

[B27] Gibson R. L. Burns J. L. Ramsey B. W. (2003). Pathophysiology and Management of Pulmonary Infections in Cystic Fibrosis. Am. J. Respir. Crit. Care Med. 168, 918–951. doi: 10.1164/rccm.200304-505SO 14555458

[B28] Haaber J. Cohn M. T. Frees D. Andersen T. J. Ingmer H. (2012). Planktonic Aggregates of Staphylococcus Aureus Protect Against Common Antibiotics. PloS One 7, e41075. doi: 10.1371/annotation/08d0f2a8-0c40-4a0c-b546-0025648e73f0 22815921 PMC3399816

[B29] Hahn M. W. Moore E. R. Hofle M. G. (2000). Role of Microcolony Formation in the Protistan Grazing Defense of the Aquatic Bacterium Pseudomonas Sp. MWH1 Microb. Ecol. 39, 175–185. doi: 10.1007/s002480000026 12035094

[B30] Hall-Stoodley L. Costerton J. W. Stoodley P. (2004). Bacterial Biofilms: From the Natural Environment to Infectious Diseases. Nat. Rev. Microbiol. 2, 95–108. doi: 10.1038/nrmicro821 15040259

[B31] Harrison F. (2007). Microbial Ecology of the Cystic Fibrosis Lung. Microbiology 153, 917–923. doi: 10.1099/mic.0.2006/004077-0 17379702

[B32] Hauser A. R. Jain M. Bar-Meir M. McColley S. A. (2011). Clinical Significance of Microbial Infection and Adaptation in Cystic Fibrosis. Clin. Microbiol. Rev. 24, 29–70. doi: 10.1128/CMR.00036-10 21233507 PMC3021203

[B33] Holloway B. W. Krishnapillai V. Morgan A. F. (1979). Chromosomal Genetics of Pseudomonas. Microbiological Rev. 43, 73–102. doi: 10.1128/mr.43.1.73-102.1979 PMC281463 111024

[B34] Horsburgh M. J. Aish J. L. White I. J. Shaw L. Lithgow J. K. Foster S. J. (2002). sigmaB Modulates Virulence Determinant Expression and Stress Resistance: Characterization of a Functional rsbU Strain Derived From Staphylococcus Aureus 8325-4. J. Bacteriol. 184, 5457–5467. doi: 10.1128/JB.184.19.5457-5467.2002 12218034 PMC135357

[B35] Irie Y. Borlee B. R. O'Connor J. R. Hill P. J. Harwood C. S. Wozniak D. J.. (2012). Self-Produced Exopolysaccharide Is a Signal That Stimulates Biofilm Formation in Pseudomonas Aeruginosa. Proc. Natl. Acad. Sci. U. S. A. 109, 20632–20636. doi: 10.1073/pnas.1217993109 23175784 PMC3528562

[B36] Jennings L. K. Dreifus J. E. Reichhardt C. Storek K. M. Secor P. R. Wozniak D. J.. (2021). Pseudomonas Aeruginosa Aggregates in Cystic Fibrosis Sputum Produce Exopolysaccharides That Likely Impede Current Therapies. Cell Rep. 34, 108782. doi: 10.1016/j.celrep.2021.108782 33626358 PMC7958924

[B37] Jennings L. K. Storek K. M. Ledvina H. E. Coulon C. Marmont L. S. Sadovskaya I.. (2015). Pel is a Cationic Exopolysaccharide That Cross-Links Extracellular DNA in the Pseudomonas Aeruginosa Biofilm Matrix. Proc. Natl. Acad. Sci. U. S. A. 112, 11353–11358. doi: 10.1073/pnas.1503058112 26311845 PMC4568648

[B38] Jesaitis A. J. Franklin M. J. Berglund D. Sasaki M. Lord C. I. Bleazard J. B.. (2003). Compromised Host Defense on Pseudomonas Aeruginosa Biofilms: Characterization of Neutrophil and Biofilm Interactions. J. Immunol. 171, 4329–4339. doi: 10.4049/jimmunol.171.8.4329 14530358

[B39] Jorth P. Staudinger B. J. Wu X. Hisert K. B. Hayden H. Garudathri J.. (2015). Regional Isolation Drives Bacterial Diversification Within Cystic Fibrosis Lungs. Cell Host Microbe 18, 307–319. doi: 10.1016/j.chom.2015.07.006 26299432 PMC4589543

[B40] Kharazmi A. (1991). Mechanisms Involved in the Evasion of the Host Defence by Pseudomonas Aeruginosa. Immunol. Lett. 30, 201–205. doi: 10.1016/0165-2478(91)90026-7 1757106

[B41] Kim D. Barraza J. P. Arthur R. A. Hara A. Lewis K. Liu Y.. (2020). Spatial Mapping of Polymicrobial Communities Reveals a Precise Biogeography Associated With Human Dental Caries. Proc. Natl. Acad. Sci. U. S. A. 117, 12375–12386. doi: 10.1073/pnas.1919099117 32424080 PMC7275741

[B42] Kirketerp-Moller K. Jensen P. O. Fazli M. Madsen K. G. Pedersen J. Moser C.. (2008). Distribution, Organization, and Ecology of Bacteria in Chronic Wounds. J. Clin. Microbiol. 46, 2717–2722. doi: 10.1128/JCM.00501-08 18508940 PMC2519454

[B43] Kragh K. N. Alhede M. Jensen P. O. Moser C. Scheike T. Jacobsen C. S.. (2014). Polymorphonuclear Leukocytes Restrict Growth of Pseudomonas Aeruginosa in the Lungs of Cystic Fibrosis Patients. Infect. Immun. 82, 4477–4486. doi: 10.1128/IAI.01969-14 25114118 PMC4249348

[B44] LeRoux M. De Leon J. A. Kuwada N. J. Russell A. B. Pinto-Santini D. Hood R. D.. (2012). Quantitative Single-Cell Characterization of Bacterial Interactions Reveals Type VI Secretion Is a Double-Edged Sword. Proc. Natl. Acad. Sci. U. S. A. 109, 19804–19809. doi: 10.7554/eLife.05701 23150540 PMC3511723

[B45] LeRoux M. Kirkpatrick R. L. Montauti E. I. Tran B. Q. Peterson S. B. Harding B. N.. (2015). Kin Cell Lysis Is a Danger Signal That Activates Antibacterial Pathways of Pseudomonas Aeruginosa. Elife 4, 3744–3753. doi: 10.7554/eLife.05701.032 PMC4348357 25643398

[B46] Li W. Y. Palis H. Merindol R. Majimel J. Ravaine S. Duguet E. (2020). Colloidal Molecules and Patchy Particles: Complementary Concepts, Synthesis and Self-Assembly. Chem. Soc. Rev. 49, 1955–1976. doi: 10.1039/C9CS00804G 32108182

[B47] Lory S. Jin S. Boyd J. M. Rakeman J. L. Bergman P. (1996). Differential Gene Expression by Pseudomonas Aeruginosa During Interaction With Respiratory Mucus. Am. J. Respir. Crit. Care Med. 154, S183–S186. doi: 10.1164/ajrccm/154.4_Pt_2.S183 8876539

[B48] Malone C. L. Boles B. R. Lauderdale K. J. Thoendel M. Kavanaugh J. S. Horswill A. R. (2009). Fluorescent Reporters for Staphylococcus Aureus. J. Microbiol. Methods 77, 251–260. doi: 10.1016/j.mimet.2009.02.011 19264102 PMC2693297

[B49] Mann E. E. Wozniak D. J. (2012). Pseudomonas Biofilm Matrix Composition and Niche Biology. FEMS Microbiol. Rev. 36, 893–916. doi: 10.1111/j.1574-6976.2011.00322.x 22212072 PMC4409827

[B50] Marenduzzo D. Finan K. Cook P. R. (2006). The Depletion Attraction: An Underappreciated Force Driving Cellular Organization. J. Cell Biol. 175, 681–686. doi: 10.1083/jcb.200609066 17145959 PMC2064666

[B51] Mashburn L. M. Jett A. M. Akins D. R. Whiteley M. (2005). Staphylococcus Aureus Serves as an Iron Source for Pseudomonas Aeruginosa During *In Vivo* Coculture. J. Bacteriol. 187, 554–566. doi: 10.1128/JB.187.2.554-566.2005 15629927 PMC543556

[B52] Mathee K. Ciofu O. Sternberg C. Lindum P. W. Campbell J. I. A. Jensen P.. (1999). Mucoid Conversion of Pseudomonas Aeruginosa by Hydrogen Peroxide: A Mechanism for Virulence Activation in the Cystic Fibrosis Lung. Microbiology 145 (Pt 6), 1349–1357. doi: 10.1099/13500872-145-6-1349 10411261

[B53] Mavrodi D. V. Bonsall R. F. Delaney S. M. Soule M. J. Phillips G. Thomashow L. S. (2001). Functional Analysis of Genes for Biosynthesis of Pyocyanin and Phenazine-1-Carboxamide From Pseudomonas Aeruginosa PAO1. J. Bacteriol. 183, 6454–6465. doi: 10.1128/JB.183.21.6454-6465.2001 11591691 PMC100142

[B54] McFarlane N. L. Wagner N. J. Kaler E. W. Lynch M. L. (2010). Poly(ethylene Oxide) (PEO) and Poly(Vinyl Pyrolidone) (PVP) Induce Different Changes in the Colloid Stability of Nanoparticles. Langmuir 26, 13823–13830. doi: 10.1021/la101907s 20684552

[B55] Mougous J. D. Cuff M. E. Raunser S. Shen A. Zhou M. Gifford C. A.. (2006). A Virulence Locus of Pseudomonas Aeruginosa Encodes a Protein Secretion Apparatus. Science 312, 1526–1530. doi: 10.1126/science.1128393 16763151 PMC2800167

[B56] O'Toole G. Kaplan H. B. Kolter R. (2000). Biofilm Formation as Microbial Development. Annu. Rev. Microbiol. 54, 49–79. doi: 10.1146/annurev.micro.54.1.49 11018124

[B57] Palmer K. L. Mashburn L. M. Singh P. K. Whiteley M. (2005). Cystic Fibrosis Sputum Supports Growth and Cues Key Aspects of Pseudomonas Aeruginosa Physiology. J. Bacteriol. 187, 5267–5277. doi: 10.1128/JB.187.15.5267-5277.2005 16030221 PMC1196007

[B58] Pedersen S. S. Hoiby N. Espersen F. Koch C. (1992). Role of Alginate in Infection With Mucoid Pseudomonas Aeruginosa in Cystic Fibrosis. Thorax 47, 6–13. doi: 10.1136/thx.47.1.6 1539148 PMC463537

[B59] Peters V. F. D. Vis M. Tuinier R. Lekkerkerker H. N. W. (2021). Phase Separation in Mixed Suspensions of Bacteria and Nonadsorbing Polymers. J. Chem. Phys. 154, 151101. doi: 10.1063/5.0045435 33887938

[B60] Poon W. C. K. (2002). The Physics of a Model Colloid-Polymer Mixture. J. Physics-Condensed Matter 14, R859–R880. doi: 10.1088/0953-8984/14/33/201

[B61] Preska Steinberg A. Datta S. S. Naragon T. Rolando J. C. Bogatyrev S. R. Ismagilov R. F. (2019). High-Molecular-Weight Polymers From Dietary Fiber Drive Aggregation of Particulates in the Murine Small Intestine. Elife 8, 1–33. doi: 10.7554/eLife.40387 PMC6342521 30666958

[B62] Pritchett C. L. Little A. S. Okkotsu Y. Frisk A. Cody W. L. Covey C. R.. (2015). Expression Analysis of the Pseudomonas Aeruginosa AlgZR Two-Component Regulatory System. J. Bacteriol 197, 736–748. doi: 10.1128/JB.02290-14 25488298 PMC4334192

[B63] Schuster M. Greenberg E. P. (2006). A Network of Networks: Quorum-Sensing Gene Regulation in Pseudomonas Aeruginosa. Int. J. Med. Microbiol. IJMM 296, 73–81. doi: 10.1016/j.ijmm.2006.01.036 16476569

[B64] Schwab U. Abdullah L. H. Perlmutt O. S. Albert D. Davis C. W. Arnold R. R.. (2014). Localization of Burkholderia Cepacia Complex Bacteria in Cystic Fibrosis Lungs and Interactions With Pseudomonas Aeruginosa in Hypoxic Mucus. Infect. Immun. 82, 4729–4745. doi: 10.1128/IAI.01876-14 25156735 PMC4249344

[B65] Schwarz-Linek J. Dorken G. Winkler A. Wilson L. G. Pham N. T. French C. E.. (2010). Polymer-Induced Phase Separation in Suspensions of Bacteria. Epl 89, 68003–68014. doi: 10.1209/0295-5075/89/68003

[B66] Schwarz-Linek J. Valeriani C. Cacciuto A. Cates M. E. Marenduzzo D. Morozov A. N.. (2012). Phase Separation and Rotor Self-Assembly in Active Particle Suspensions. Proc. Natl. Acad. Sci. U. S. A. 109, 4052–4057. doi: 10.1073/pnas.1116334109 22392986 PMC3306685

[B67] Schwarz-Linek J. Winkler A. Wilson L. G. Pham N. T. Schilling T. Poon W. C. K. (2010). Polymer-Induced Phase Separation in Escherichia Coli Suspensions. Soft Matter 6, 4540–4549. doi: 10.1039/c0sm00214c

[B68] Secor P. R. Michaels L. A. Ratjen A. Jennings L. K. Singh P. K. (2018). Entropically Driven Aggregation of Bacteria by Host Polymers Promotes Antibiotic Tolerance in Pseudomonas Aeruginosa. Proc. Natl. Acad. Sci. U. S. A 115 (42), 10780–10785. doi: 10.1073/pnas.1806005115 30275316 PMC6196481

[B69] Siehnel R. Traxler B. An D. D. Parsek M. R. Schaefer A. L. Singh P. K. (2010). A Unique Regulator Controls the Activation Threshold of Quorum-Regulated Genes in Pseudomonas Aeruginosa. Proc. Natl. Acad. Sci. U. S. A. 107, 7916–7921. doi: 10.1073/pnas.0908511107 20378835 PMC2867882

[B70] Singh P. K. Schaefer A. L. Parsek M. R. Moninger T. O. Welsh M. J. Greenberg E. P. (2000). Quorum-Sensing Signals Indicate That Cystic Fibrosis Lungs are Infected With Bacterial Biofilms. Nature 407, 762–764. doi: 10.1038/35037627 11048725

[B71] Smith E. E. Buckley D. G. Wu Z. Saenphimmachak C. Hoffman L. R. D'Argenio D. A.. (2006). Genetic Adaptation by Pseudomonas Aeruginosa to the Airways of Cystic Fibrosis Patients. Proc. Natl. Acad. Sci. U. S. A. 103, 8487–8492. doi: 10.1021/ac051437y 16687478 PMC1482519

[B72] Sonderholm M. Kragh K. N. Koren K. Jakobsen T. H. Darch S. E. Alhede M.. (2017). Pseudomonas Aeruginosa Aggregate Formation in an Alginate Bead Model System Exhibits *In Vivo-*Like Characteristics. Appl. Environ. Microbiol. 83, e00113-17. doi: 10.1128/AEM.00113-17 28258141 PMC5394317

[B73] Speare L. Smith S. Salvato F. Kleiner M. Septer A. N. (2020). Environmental Viscosity Modulates Interbacterial Killing During Habitat Transition. mBio 11, e03060-19. doi: 10.1128/mBio.03060-19 32019799 PMC7002345

[B74] Stacy A. McNally L. Darch S. E. Brown S. P. Whiteley M. (2016). The Biogeography of Polymicrobial Infection. Nat. Rev. Microbiol. 14, 93–105. doi: 10.1038/nrmicro.2015.8 26714431 PMC5116812

[B75] Starkey M. Hickman J. H. Ma L. Zhang N. De Long S. Hinz A.. (2009). Pseudomonas Aeruginosa Rugose Small-Colony Variants Have Adaptations That Likely Promote Persistence in the Cystic Fibrosis Lung. J. bacteriology 191, 3492–3503. doi: 10.1128/JB.00119-09 PMC2681918 19329647

[B76] Staudinger B. J. Muller J. F. Halldorsson S. Boles B. Angermeyer A. Nguyen D.. (2014). Conditions Associated With the Cystic Fibrosis Defect Promote Chronic Pseudomonas Aeruginosa Infection. Am. J. Respir. Crit. Care Med. 189, 812–824. doi: 10.1164/rccm.201312-2142OC 24467627 PMC4225830

[B77] Stewart P. S. Costerton J. W. (2001). Antibiotic Resistance of Bacteria in Biofilms. Lancet 358, 135–138. doi: 10.1016/S0140-6736(01)05321-1 11463434

[B78] Varga J. J. Losada L. Zelazny A. M. Kim M. McCorrison J. Brinkac L.. (2013). Draft Genome Sequences of Burkholderia Cenocepacia ET12 Lineage Strains K56-2 and BC7. Genome Announc 1, e00841-13. doi: 10.1128/genomeA.00841-13 24136849 PMC3798455

[B79] Wang J. Lory S. Ramphal R. Jin S. (1996). Isolation and Characterization of Pseudomonas Aeruginosa Genes Inducible by Respiratory Mucus Derived From Cystic Fibrosis Patients. Mol. Microbiol. 22, 1005–1012. doi: 10.1046/j.1365-2958.1996.01533.x 8971720

[B80] Wiens J. R. Vasil A. I. Schurr M. J. Vasil M. L. (2014). Iron-Regulated Expression of Alginate Production, Mucoid Phenotype, and Biofilm Formation by Pseudomonas Aeruginosa. mBio 5, e01010–e01013. doi: 10.1128/mBio.01010-13 24496793 PMC3950519

[B81] Wilpiszeski R. L. Aufrecht J. A. Retterer S. T. Sullivan M. B. Graham D. E. Pierce E. M.. (2019). Soil Aggregate Microbial Communities: Towards Understanding Microbiome Interactions at Biologically Relevant Scales. Appl. Environ. Microbiol. 85, e00324-19. doi: 10.1128/AEM.00324-19 31076430 PMC6606860

[B82] Wood L. F. Ohman D. E. (2009). Use of Cell Wall Stress to Characterize Sigma 22 (AlgT/U) Activation by Regulated Proteolysis and its Regulon in Pseudomonas Aeruginosa. Mol. Microbiol. 72, 183–201. doi: 10.1111/j.1365-2958.2009.06635.x 19226327

[B83] Worlitzsch D. Tarran R. Ulrich M. Schwab U. Cekici A. Meyer K. C.. (2002). Effects of Reduced Mucus Oxygen Concentration in Airway Pseudomonas Infections of Cystic Fibrosis Patients. J. Clin. Invest. 109, 317–325. doi: 10.1172/JCI0213870 11827991 PMC150856

[B84] Yu Y. Kim H. S. Chua H. H. Lin C. H. Sim S. H. Lin D.. (2006). Genomic Patterns of Pathogen Evolution Revealed by Comparison of Burkholderia Pseudomallei, the Causative Agent of Melioidosis, to Avirulent Burkholderia Thailandensis. BMC Microbiol. 6, 46. doi: 10.1186/1471-2180-6-46 16725056 PMC1508146

[B85] Zhao K. Tseng B. S. Beckerman B. Jin F. Gibiansky M. L. Harrison J. J.. (2013). Psl Trails Guide Exploration and Microcolony Formation in Pseudomonas Aeruginosa Biofilms. Nature 497, 388–391. doi: 10.1038/nature12155 23657259 PMC4109411

