# Supplementary figures and images for: The Depletion Mechanism Actuates Bacterial Aggregation by Exopolysaccharides and Determines Species Distribution & Composition in Bacterial Aggregates
Source: Front Cell Infect Microbiol. 2022 Jun 16;12:869736. doi: 10.3389/fcimb.2022.869736 (PMC9243289; doi:10.3389/fcimb.2022.869736)

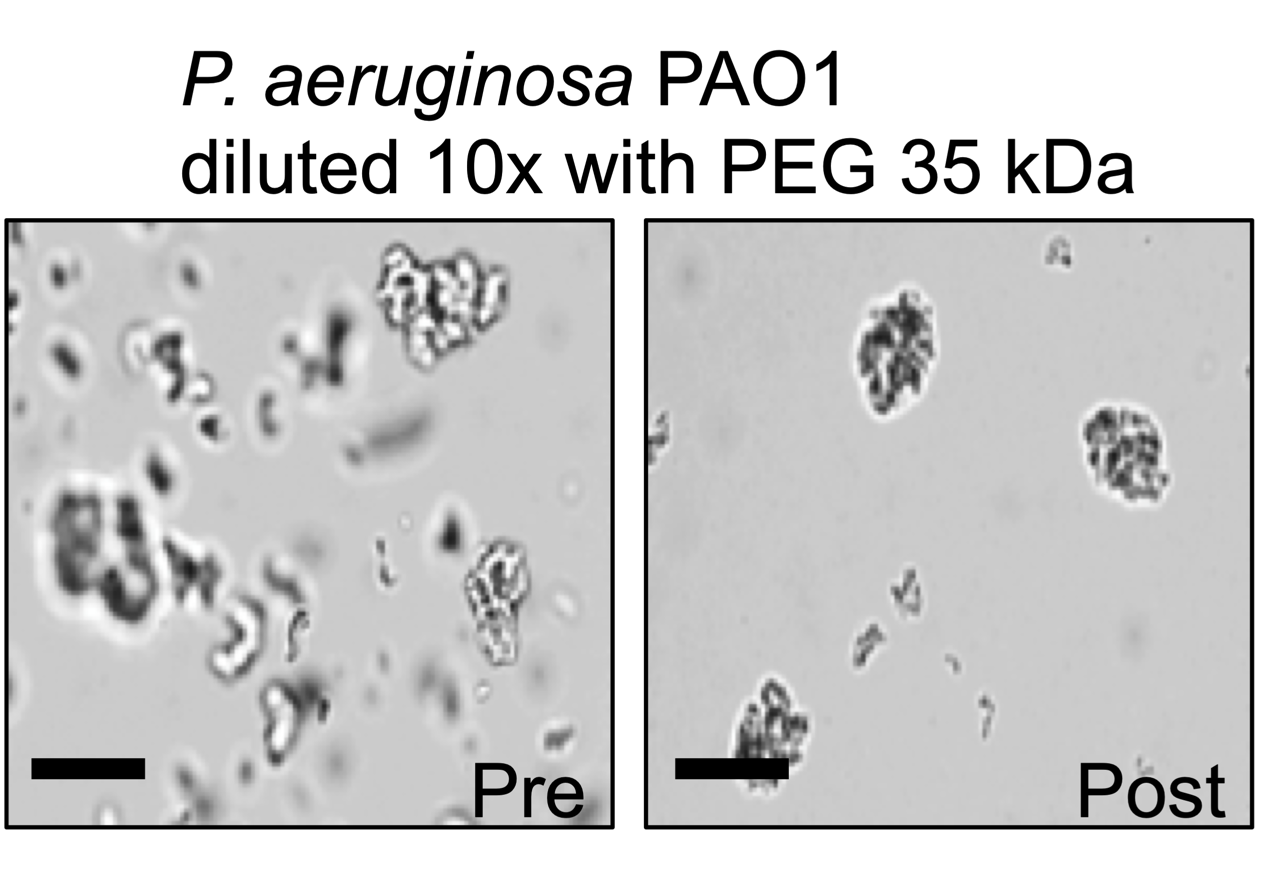

Supplement: Supplementary Figure 1 — Depletion aggregation was induced with 30% w/vol PEG 35 kDa for 18 hours. P. aeruginosa PAO1 depletion aggregates were then diluted 10X with additional PEG 35 kDa. Scale bar 40 µm. [file Image_1.tiff]

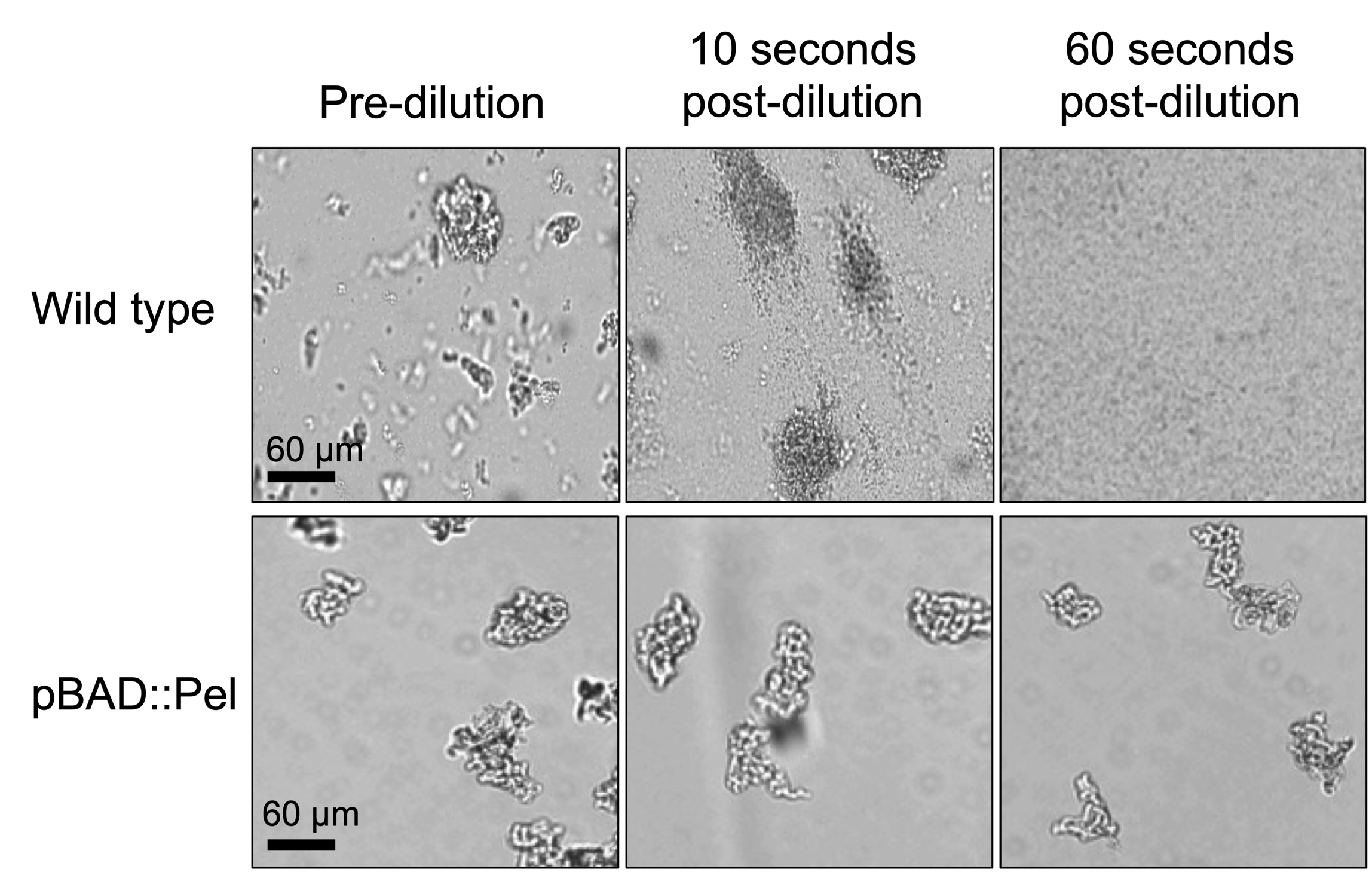

Supplement: Supplementary Figure 2 — Depletion aggregate dispersal phenotypes of P. aeruginosa PAO1 and PAO1 pBAD::Pel. Aggregate dispersal of wild-type and pBAD::Pel strains was measured. Depletion aggregation was induced with 30% w/vol PEG 35 kDa for 18 hours. Depletion aggregates were then diluted 10X with PBS and representative images were acquired pre-dilution and 10- and 60-seconds post-dilution. [file Image_2.tiff]

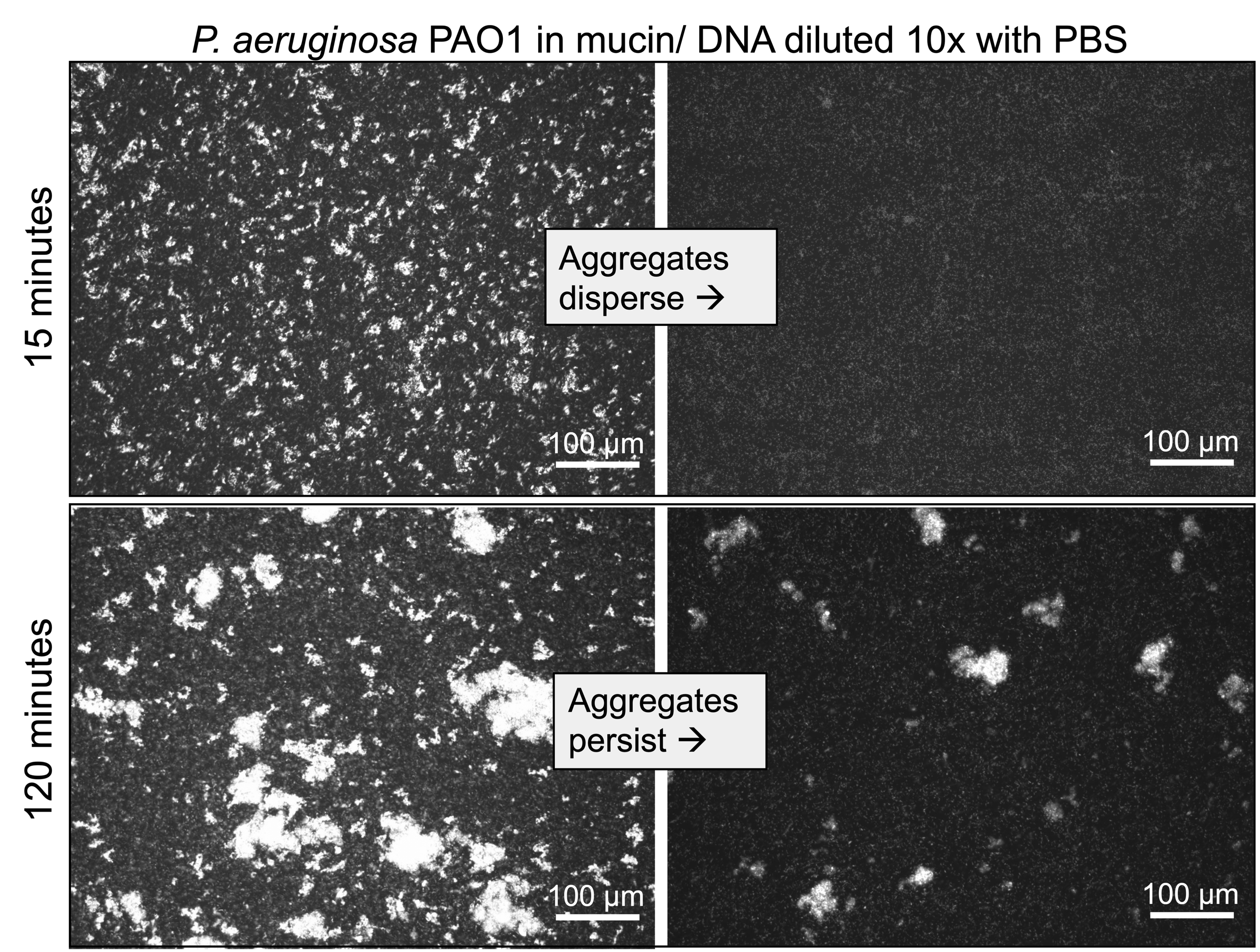

Supplement: Supplementary Figure 3 — Depletion aggregate dispersal phenotypes of P. aeruginosa in mixtures of mucin and DNA. Aggregate dispersal of fluorescent P. aeruginosa PAO1 was measured. Depletion aggregation was induced with 4% w/vol mucin and 2 mg/ml DNA for 15 or 120 minutes. Depletion aggregates were then diluted 10X with PBS and images were acquired immediately pre- and immediately post-dilution. Representative images are shown. [file Image_3.tiff]

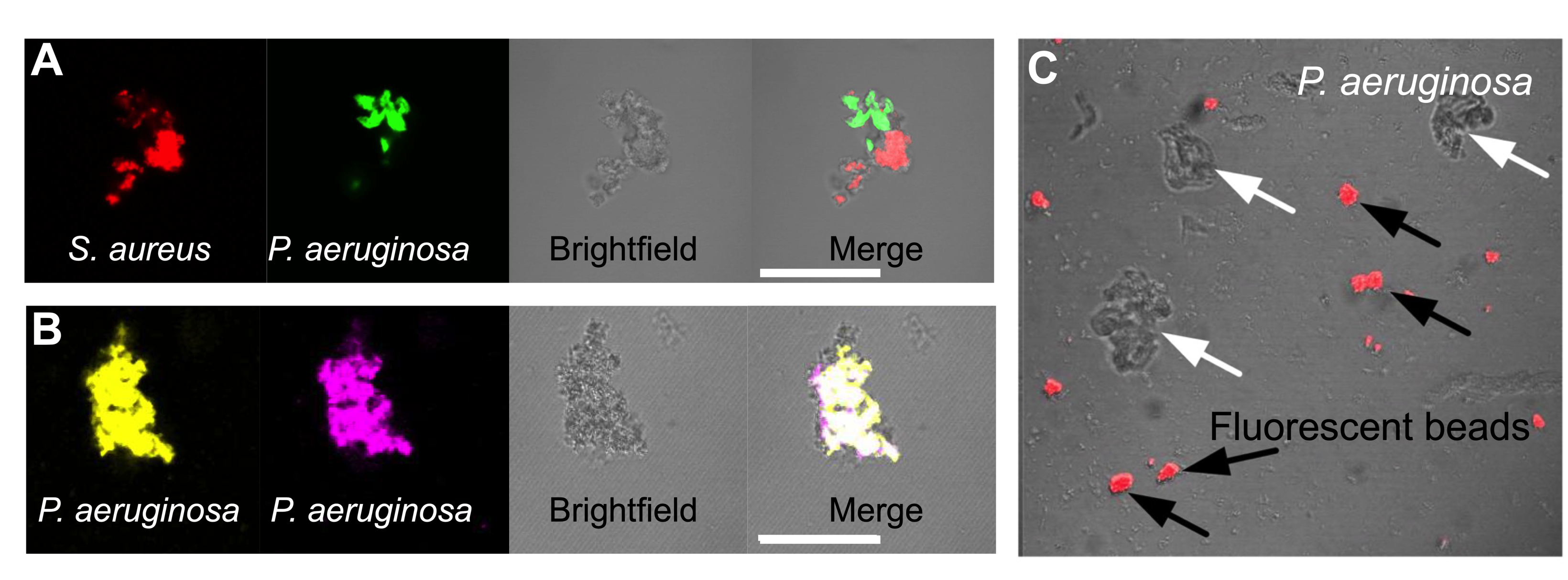

Supplement: Supplementary Figure 4 — Depletion aggregation operates on dead cells and inert latex beads. (A, B) Depletion aggregation was induced with 30% w/vol PEG 35 kDa using combinations of the indicated species of dead formalin-fixed bacteria. Fluorescent microscopy was used to image aggregates after 18-h of growth. Bar, 30 µm. (C) P. aeruginosa (white arrows) and fluorescent spherical latex beads (2 µm diameter, black arrows) were aggregated using 30% w/vol PEG 35 kDa for 18-h and imaged using fluorescent and brightfield microscopy. Bar, 30 µm. [file Image_4.tiff]

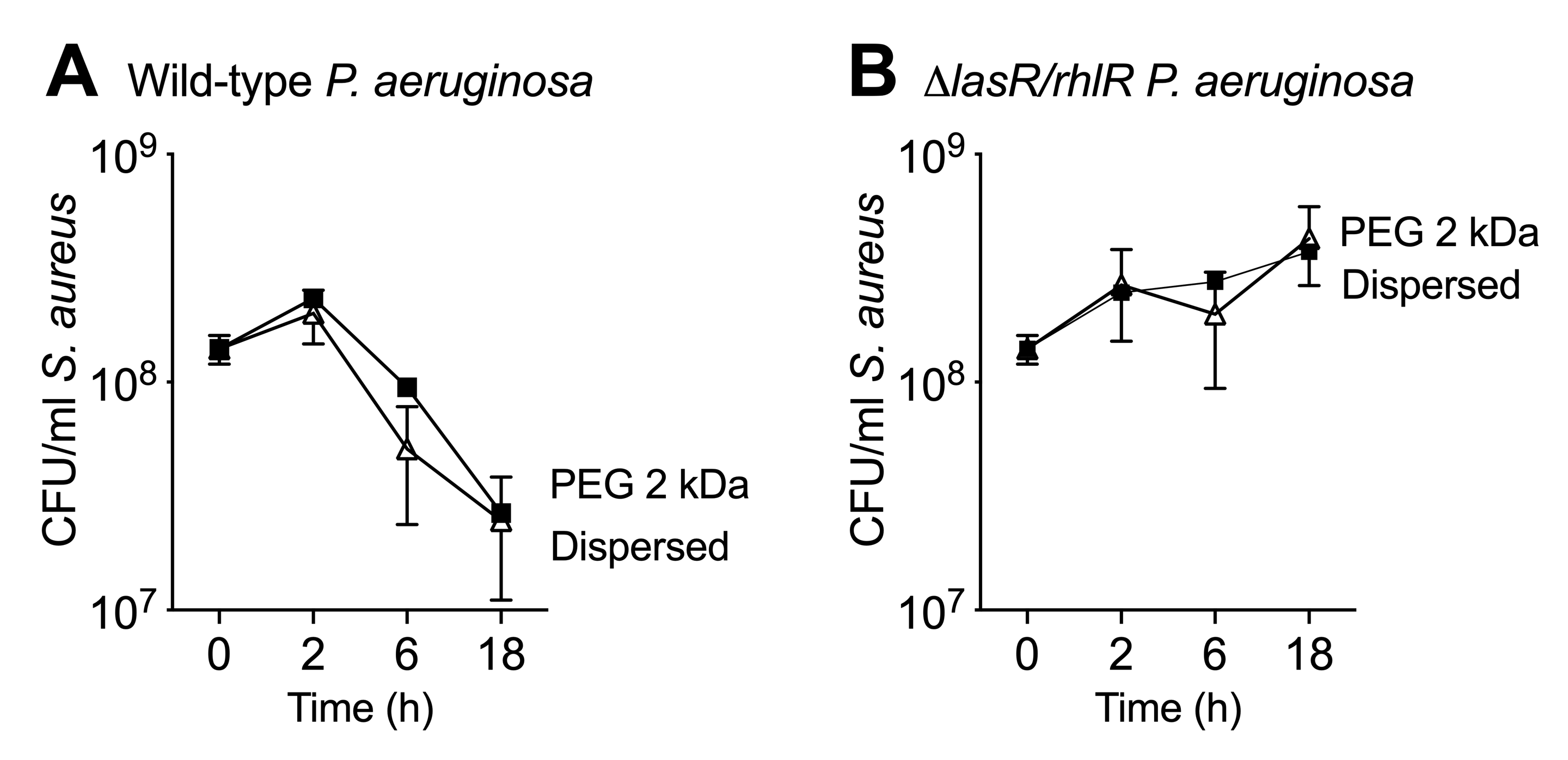

Supplement: Supplementary Figure 5 — PEG does not inactivate antimicrobials present in P. aeruginosa supernatants. One possible explanation for the reduced killing of aggregated S. aureus (see ) was that PEG somehow inactivated antimicrobials present in wild-type P. aeruginosa supernatants. To address this possibility, we used a lower molecular weight PEG (PEG 2 kDa). As polymer molecular weight decreases, the polymer concentration required to induce depletion aggregation of a given number of cells increases. Thus, PEG 2 kDa does not promote depletion aggregation at 30% w/vol. Dissolving PEG 2 kDa into wild-type P. aeruginosa supernatants did not affect S. aureus inhibition in supernatants collected from (A) wild-type or (B) ΔlasR/rhlR overnight cultures compared to polymer-free controls, indicating that PEG did not inactivate antimicrobials present in P. aeruginosa supernatants. [file Image_5.tiff]
